# A novel NMDA receptor modulator: the antidepressant effect and mechanism of GW043

**DOI:** 10.1111/cns.14598

**Published:** 2024-02-08

**Authors:** Murezati Tiliwaerde, Nana Gao, Yaqi Yang, Zengliang Jin

**Affiliations:** ^1^ Department of Pharmacology, School of Basic Medical Sciences Capital Medical University Beijing China; ^2^ Department of Gastrointestinal Surgery and Clinical Nutrition, Beijing Shijitan Hospital Captial Medical University Beijing China; ^3^ Department of Pharmacy, Beijing Chaoyang Hospital Capital Medical University Beijing China

**Keywords:** antidepressant, depression, NMDAR, partial agonist

## Abstract

**Aims:**

The *N*‐methyl‐d‐aspartate (NMDA) receptor (NMDAR) has been proven to be strongly correlated with rapid antidepressant effects. Here, GW043, as a new compound targeting NMDAR, we explored its antidepressant effects and its mechanism of action.

**Methods:**

Our study utilized electrophysiological techniques to confirm the effect of GW043 on NMDAR currents. Additionally, we assessed the selectivity of GW043 through high‐throughput receptor‐ligand binding experiments. The antidepressant properties of GW043 were examined using rodent behavioral models including the Forced Swim Test (FST), Tail Suspension Test (TST), and Chronic Unpredictable Mild Stress (CUMS). Mechanistic insight into GW043's onset was gained through western blot analysis, BrdU staining, Golgi staining, and electrophysiological techniques.

**Results:**

Electrophysiological studies indicated that GW043 acts as a partial agonist of NMDAR. Behavioral experiments confirmed the antidepressant effect of GW043 in rodents. Mechanistic investigations revealed that GW043 modulates synaptic plasticity through the LTP and BDNF‐mTOR pathways, consequently leading to an increase in the number of newborn neurons and subsequent antidepressant effects.

**Conclusion:**

Our findings disclose that GW043, as a partial agonist of NMDAR, can reverse depression‐like behaviors in rats by modulating synaptic plasticity, indicating its potential as an antidepressant agent.

## Graphical abstract

Oral administration of GW043 enhances LTP and activates the BDNF‐mTOR pathway, thereby modulating synaptic plasticity and showing significant antidepressant effects in animal models of depression.

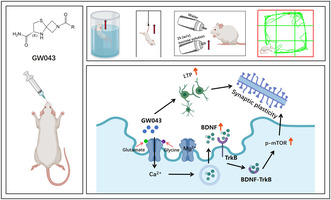

## 1 INTRODUCTION

Psychological health disorders have emerged as prominent contributors to global disability, giving rise to significant health and socioeconomic challenges. Depression, a cluster of psychiatric disorders marked by a despondent mood, diminished pleasure, decreased volitional engagement, and concomitant cognitive impairment, affects an estimated 350 million individuals worldwide.^1^, ^2^ While medication remains the primary approach for managing depression, conventional antidepressants like tricyclic antidepressants (TCAs) and selective serotonin reuptake inhibitors (SSRIs) suffer from drawbacks such as delayed efficacy, sexual dysfunction, and limited remission rates.

Along with intensive research of the mechanism of depression, NMDAR, a heterotetrameric complex expressing multiple subtypes with unique properties, were shown to play a critical role in the regulation of normal neuronal function, including activity‐dependent synaptic plasticity related to mood, learning and memory.^3^ NMDAR have been reported to be strongly associated with depression and to mediate rapid antidepressant effects,^4^ compared to traditional antidepressants. Ketamine, a competitive antagonist of the *N*‐methyl‐d‐aspartate receptor (NMDAR), has demonstrated efficacy in the treatment of depression, including treatment‐resistant depression and bipolar depression, exhibiting both rapid and sustained therapeutic effects.^5^, ^6^ The action of ketamine has been reported to derepress glutamate release by blocking the NMDAR of the gamma aminobutyric acid (GABA) interneuron, which in turn activates the mammalian target of rapamycin protein (mTOR) signaling pathway.^7^ Additionally, ketamine‐induced effects were associated with dephosphorylation of eEF2 and enhanced BDNF synthesis and affected lateral habenula neuron function.^8^ Notwithstanding the disparities in ketamine's two mechanisms, several studies propose that the augmentation of BDNF synthesis, along with the activation of BDNF‐dependent signaling pathways, could serve as uniform molecular targets by which ketamine exerts its antidepressant effects.^9^, ^10^

Ketamine (R,S‐ketamine) is a racemic mixture that exhibits different affinities for NMDAR with each enantiomer (R and S‐ketamine enantiomers), specifically S‐ketamine (*K*_i_ = 0.3 μm), R,S‐ketamine (*K*_*i*_ = 0,53 μm), and R‐ketamine (*K*_i_ = 1.4 μm), respectively.^11^ Both isomers exhibited antidepressant effects, but experimental data suggest that (R)‐ketamine exhibited more powerful antidepressant effects^12^ and lower side effects than (S)‐ketamine and (R,S)‐ketamine.^13^, ^14^ Also, R‐ketamine has been reported to be effective in bipolar disorder.^15^ Following clinical trial evaluation, on March 5, 2019, the U.S. Food and Drug Administration (FDA) approved Janssen Pharmaceuticals' Spravato (esketamine, S‐ketamine) nasal spray in combination with an oral antidepressant for use in refractory depression with risk of suicide. Notably, this was the landmark endorsement by the FDA for the use of any compounds in the ketamine series for therapeutic purposes. Widely known, ketamine is limited by proposed psychiatric side effects such as perceptual disturbances, strange or abnormal sensations, and addictive applications.^16^, ^17^ Although Spravato achieved a remission rate of more than 50% and 76.5% of patients were still responding at the end of 44 weeks of long‐term treatment, it did not completely resolve the serious adverse effects of dissociation, abuse/addictive potential, and some patients also had adverse events such as aggression, sedation, disorientation, and suicidal ideation.^18^

The past decade has also seen numerous NMDAR antagonists enter clinical trials. Dextromethorphan, a non‐opioid derivative, prominently utilized as a centrally acting cough suppressant, primarily functions by curtailing the cough reflex. Additionally, it acts as a non‐competitive antagonist of NMDA receptors. Its combination with quinidine (phase II) or bupropion (phase III) increases bioavailability.^19^, ^20^ Dextromethorphan/bupropion approved in the U.S. for the treatment of adults with major depression disorder (MDD) in August 2022.^21^ Absolutely, nitrous oxide and dextromethadone, which are known to inhibit NMDAR, as well as the GluN2B subtype‐selective NMDAR antagonists EVT‐101 and MIJ821, have all been the subjects of clinical trials. However, the final outcomes of these investigations are yet to be released or are unknown at this time.^22^ Despite some safety considerations associated with rapidly acting antidepressants derived from NMDAR antagonists, escalating evidence indicates a considerable potential for NMDAR in the therapeutic management of depression. Furthermore, prior mechanistic investigations have elucidated the prompt initiation of NMDAR antagonists' effects on BDNF‐mediated biochemical processes.^22^, ^23^

Curiously, rapastinel (GLYX‐13), a partial agonist of NMDAR‐NR2B, demonstrated swift and minimal side‐effect antidepressant properties.^24^, ^25^ In the double‐blind, randomized, placebo‐controlled study, a single intravenous (i.v) dose of rapastinel (1, 5, 10 or 30 mg/kg) reduced depressive symptoms within 2 h and this effect lasted for an average of 7 days. The mechanism of the antidepressant properties of rapastinel is unclear, and the prevailing explanation is that it notably enhances long‐term potentiation (LTP) and induces synaptic plasticity through the role of BDNF, which a possible reason for the long‐term antidepressant effect.^26^, ^27^, ^28^ Unfortunately, the Phase III clinical trial for rapastinel was ultimately terminated during subsequent development due to its lower effectiveness compared to the placebo.

GW043 is a small molecule compound that we have designed and synthesized to target NMDAR. At present, it is undergoing phase I clinical trials. In this study, we confirmed its antidepressant properties and the mechanism of operation in rodents, using electrophysiological experiments, pharmacodynamic experiments, and biochemical tests.

## 2 MATERIALS AND METHODS

### 2.1 Animals

SD rats (190 ± 10 g), Wistar rats (165 ± 15 g), and ICR mice (20 ± 2 g) were provided by the Laboratory Animal Section of Capital Medical University (Beijing, China). Animals were housed in groups at constant room temperature (23 ± 1°C), humidity (50–60%), and under a 12 h:12 h light/dark cycle (lightingat 8:00). Food and water were available at all times. All procedures were in accordance with the Guide for the Care and Use of Laboratory Animals issued by the National Institutes of Health and approved by the Animal Care and Use Committee of Capital Medical University (approval number SCXK‐2016‐0002). To reduce the number of laboratory animals and alleviate their suffering with each attempt.

### 2.2 Drugs

**FIGURE 1:**
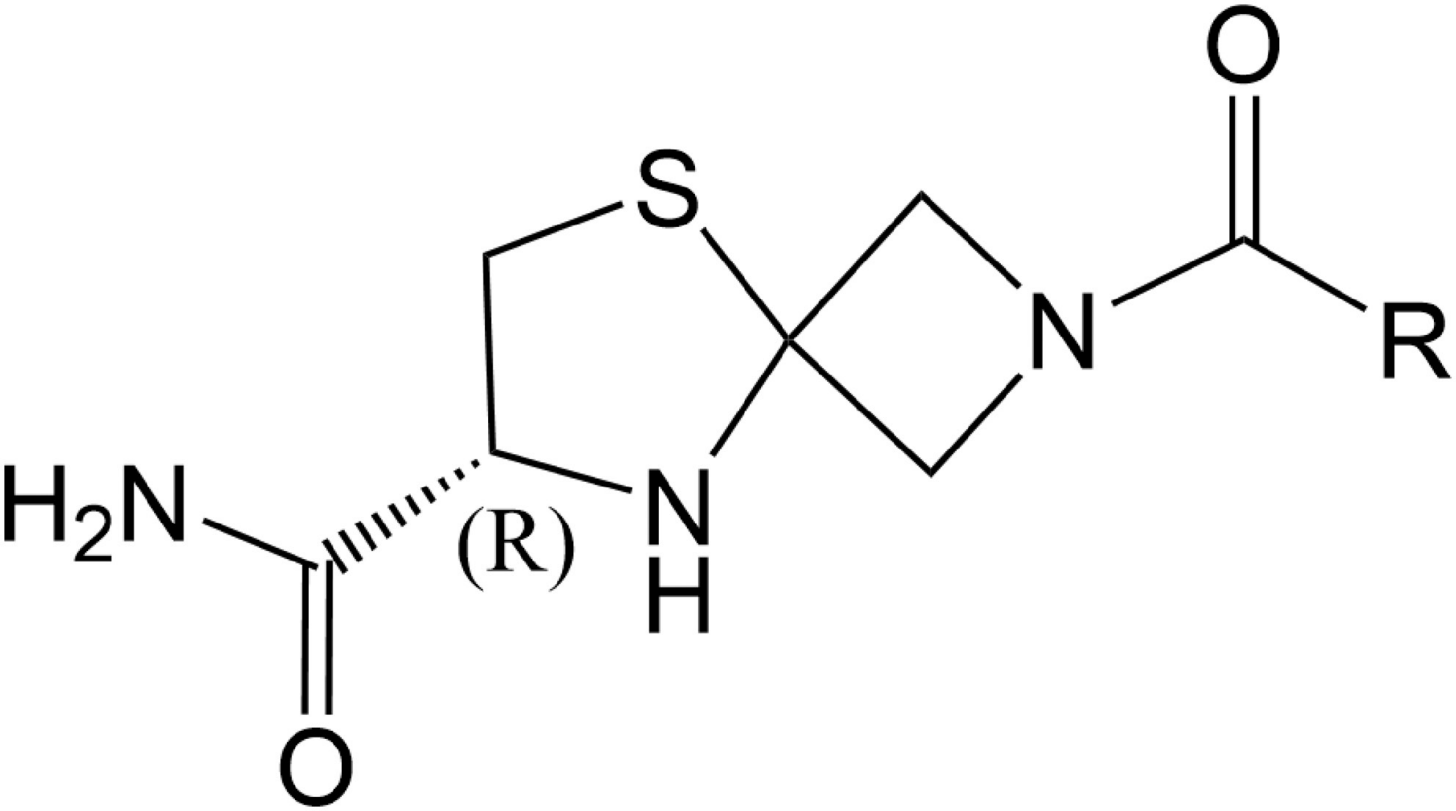
Chemical structure of GW043.

Compound GW043 (molecular weight: 310.8) from Beijing GuangWei Pharmaceutical Technology Co, and the structure is presented in Figure 1; GW043 and fluoxetine were dissolved in distilled water, which was provided by Capital Medical University; methyllycaconitine (MLA) purchased from MedChemExpress (HY‐N2332A); polyethyleneimine (PEI) purchased from Sigma‐Aldrich (PVP360); bovine serum albumin (BSA) purchased from Huaxingbio (HX3303); phenylmethylsulfonyl fluoride (PMSF) purchased from Sigma‐Aldrich (P7626); serotonin hydrochloride (5‐TH) purchased from Sigma‐Aldrich (H9523); scintillation cocktail purchased from PerkinElmer (1200‐439); dimethyl sulfoxide (DMSO) purchased from Sigma (D4540); artificial cerebrospinal fluid (ACSF) purchased from Phygene (PH1851); NaCl purchased from Sigma (S5886); KCl purchased from Sigma (P5405); MgSO_4_ purchased from Sigma (M2643); D‐Glucose purchased from Sigma (G8270); CaCl_2_∙2H_2_O purchased from Sigma (C7902); NaHCO_3_ purchased from Sigma (S5761); NaH_2_PO_4_∙2H_2_O purchased from GENRAL‐REAGENT (G21298B); sucrose purchased from SCR (10021418); MgCl_2_∙6H_2_O purchased from Sigma (M2393); Cs‐MeSO_3_ purchased from Sigma (C1426); HEPES purchased from Santa Cruz (sc‐29097A); Na_2−_GTP purchased from ACROS (226250500); Mg‐ATP purchased from Sigma (A9187); EGTA purchased from Sigma (E3889); glycine purchased from Sigma (G7126); glumate purchased from Sigma (G1251); FD Rapid GolgiStain™ Kit (PK401); Thermo BCA Protein Assay Kit (23227); anti‐BDNF antibody (ab108319); GAPDH rabbit polyclonal antibody (10494‐1‐AP); mTOR mouse monoclonal antibody (66888‐1‐lg); BrdU Mouse mAb (CST #5292); phospho‐mTOR Rabbit mAb (CST #5536); extra range prestained protein marker (proteintech PL00003); goat anti‐rabbit IgG (YEASEN HB190419); goat Anti‐Mouse IgG H&L (Alexa Fluor 488) (ab150113).

### 2.3 Screening for other receptors in a binding assays

The following receptors were screened by high‐throughput binding assays: A_1_, A_2A_, A_3_, α_1_, α_2_, β_1_, β_2_, AT_1_, BZD (central), B_2_, CB_1_, CCK_1_ (CCK_A_), D_1_, D_2S_, ET_A_, GABA, GAL_2_, CXCR2 (IL‐8B), CCR1, H_1_, H_2_, MC_4_, MT_1_ (ML_1A_), M_1_, M_2_, M_3_, NK_2_, NK_3_, Y1, Y2, NTS1 (NT1), δ (DOP), kappa (h), μ (MOP), NOP (ORL1), EP4, 5‐HT1A, 5‐HT1B, 5‐HT2A, 5‐HT2B, 5‐HT3, 5‐HT5a, 5‐HT6, 5‐HT7, sst, VPAC1 (VIP1), V1a, Ca^2+^ channel (L, verapamil site), KV channel, SKCa channel, Na^+^ channel (site 2), Cl^−^ channel (GABA‐gated), norepinephrine transporter, dopamine transporter. For details of the experimental protocol, please refer to the supplementary document.

### 2.4 Electrophysiology

#### 2.4.1 Preparation of slices

Animals were deeply anesthetized with uratan and then decapitated (25%, 1 mL/100 g), the brains were quickly removed from the cranial cavity and transferred to artificial cerebrospinal fluid (ACSF) which contained (in mM): 234 Sucrose, 2.5 KCl, 1.25 NaH_2_PO_4_∙2H_2_O, 25 NaHCO_3_, 25 D‐Glucose, 0.5 CaCl_2_∙2H_2_O, and 10 MgSO_4_; at pH 7.2–7.4, gassed continuously with 95% O_2_/5% CO_2_. Excision of the cerebellum and frontal lobe while separating the two cerebral hemispheres. Freed hippocampus was held upright on an agar block with 502 glue as well as anchored to a specimen base that was fixed in a specimen tank containing ice‐cold sections of ACSF. Coronal sections 380 μm thick were cut with a vibrating microtome (Leica, VT 1000 S, Germany) and transferred to a saturated 32°C constant temperature water bath recording solution ACSF for 30 min incubation. Before the brain slice is transferred to the recording chamber perfused at 6 mL/min with ACSF at 31.0 ± 1°C, slices were incubated for at least 1.5 h on standby at 22–23°C.

#### 2.4.2 The effects of GW043 on NMDAR currents in CA3‐CA1 region of rat hippocampus

Capillary glass tubes were drawn into stimulation electrodes and recording electrodes with a microelectrode drawing instrument, and the stimulation electrodes were placed at a distance of about 200–400 μm from the cells. The recording electrode is given positive pressure after entering the liquid, and negative pressure is given for aspiration after touching the cell membrane to form a GΩ seal. A rapid capacitive compensation was performed and negative pressure was continued to be given to aspirate the cell membrane. Recording a 5 mV voltage stimulus after waiting for 3–5 min for cell stabilization in Gap‐free mode (test series resistance). The voltage stimulation scheme for recording NMDA currents was as follows: the membrane potential was clamped at +50 mV, appropriate stimulation intensity was given until the NMDA current reached about 100 pA. Recording was started for 10 min or stable, and the working solution of the GW043 to be tested was given for 15 min or until stable, each trace interval was 30 s. The change in series resistance was tested before and after recording.

#### 2.4.3 The effect of GW043 on the change of LTP in CA3‐CA1 region of mouse hippocampus

Mouse brain slices were prepared in the same way as above. For extracellular recordings, electrical recording by a borosilicate glass capillary tube has an outer diameter of 1.5 mm and an inner diameter of 1.10 mm, (GB 150F‐8P, Sutter Instrument, USA) of 10 cm length. An electrode puller (P97; Sutter Instrument, USA) was used to pull glass electrodes, filled with ACSF solution, and connected to a microelectrode amplifier (Axonpatch 700B; Molecular Devices). Ag‐AgCl is used as the reference electrode, placed in the CA1 region of the radiation layer. Excitatory postsynaptic field potentials (fEPSP) were excited by a glass electrodewith recording fluid ACSF placed in the CA3 region, which contained (in mM): 125 NaCl, 2.5 KCl, 1.25 NaH_2_PO_4_∙2H_2_O, 25 NaHCO_3_,10 D‐Glucose, 2 CaCl_2_∙2H_2_O, and 1.5 MgSO_4_ (gassed continuously with 95% O_2_/5% CO), with a stimulation resistance of approximately 500 kΩ and a recording resistance of approximately 1.0 MΩ. The stimulation electrode is 400 μm from the recording electrode. A single test pulse lasting 100 μs with stimulation parameters from 10 to 40 μA is run in I = 0 mode. The stimulation intensity was adjusted to find the maximum value, and the intensity of the stimulus that elicited 40%–50% of the maximum response amplitude was used as the basal stimulus intensity, with a stable response recorded for >30 min as a baseline and a fluctuation range of <10% in slope, and high‐frequency stimulation (HFS) stimulation (100 Hz per string for 1 s; three stimulations at 10 s intervals) was run immediately after the basal stimulus to induce LTP, followed by a single test pulse stimulation for 60 min.

Change in fEPSP slope over time: for each LTP response, the slope of fEPSP after stimulation was normalized by reference to the mean (100%) of the baseline fEPSP slope; data from the same time point in the experiment were taken as the mean; the extent of LTP was expressed as a percentage of the baseline fEPSP slope. Comparison between groups for long‐range synaptic plasticity: for each LTP response, the 20 signals at 50–60 min after stimulation were normalized by reference to the mean of the 20 signals 10 min before stimulation and taken as the mean; thereafter, the same group was taken as the mean.

#### 2.4.4 The effect of GW043 on NMDAR currents expressed in oocytes

##### Isolation and injection of oocytes

Xenopus oocytes were surgically harvested from mature, gravid female Xenopus laevis frogs. The oocytes were transferred to a sterile 50 mL centrifuge tube, washed repeatedly with calcium‐free ND96 solution (96 mM NaCl, 2 mM KCl, 1 mM MgCl_2_·6H_2_O, 5 mM HEPES, 2.5 mM sodium pyruvate, 0.1 mg/mL BSA, 50 mg/mL gentamicin sulfate pH = 7.4 (NaOH)) to remove residual blood, and then collagenase solution with a final concentration of 1 mg/mL was added and shaken slightly at room temperature for about 1–2 h. Mature oocytes of stages V and VI were observed and selected under a high magnification microscope. Each oocyte was injected with 35 nL of cRNA mixed rNR1 and rNR2B at a concentration of 1000 ng/μL in a volume ratio of 1:3. The injected oocytes were placed in ND‐96 culture medium and incubated in a biochemical incubator (17°C) for 48 h (requiring daily fluid changes) before being used for electrophysiological assays.

#### 2.4.5 Electrophysiological recording

The oocyte membrane was voltage‐clamped at 70 mV. Peak NMDAR currents were recorded sequentially by spraying a mixture of different concentrations of GW043 and 0.1 μM glycine and 0.5 μM L‐glutamate on the cell surface. Test data are collected by TECH LIH amplifiers (HEKA) and stored in PatchMaster (HEKA) software. Expressed oocytes were placed in a recording bath in an orthomosaic microscope with a pipette. A capillary glass tube is drawn into a recording electrode using a microelectrode puller. Under an orthotropic microscope, the recording electrode was submerged into the oocyte for recording by manipulating the manual microelectrode manipulator. The test solution and the external solution without GW043 are applied to the cells by flowing sequentially from low to high concentrations through the recording bath using gravity perfusion, and a vacuum pump is used for fluid exchange during the recording. The data were repeated for each concentration in multiple cases. The current signal is digitized for analysis.

### 2.5 Tail suspension test (TST) in mice

TST was performed as described previously.^29^ 72 ICR mice were randomly divided into six groups (*n* = 12/group): control group, 10.00 mg/kg fluoxetine, 0.02 mg/kg GW043, 0.10 mg/kg GW043, 1.00 mg/kg GW043, and 10.00 mg/kg GW043. The drug was administered via gavage 30 min prior to the initiation of the experiment. One week after adaptive feeding, the TST was conducted: the hanging tail box was 25 × 25 × 35 cm, and using the tape to hang the mouse upside down in the middle of the box, the head of the mouse was about 5 cm from the bottom of the hanging tail box. Observe for 6 min and record the time of immobility after 4 min. The criterion for mobility is that the animal stops struggling, its body hangs vertically and stays still. The experiments were fasted for 12 h before the experiment and performed 30 min after intragastric administration.

### 2.6 Forced swimming test (FST) in rats

Modified FST was performed in rats as previously described.^30^ 54 naïve SD rats were randomly divided into six groups (*n* = 9/group): control group, 10 mg/kg fluoxetine, 0.1 mg/kg GW043, 1.0 mg/kg GW043, 10.0 mg/kg GW043, and 30.0 mg/kg GW043. The drug was administered via gavage 60 min prior to the initiation of the experiment. The procedure consisted of two phases, the pretest phase and the test phase, with the use of identical cylinders and conditions (20 cm in diameter, 60 cm in height, containing 33 cm of water, temperature maintained at 25°C). At pretest period, the rats were placed in the cylinder alone for 15 min of swimming; after 24 h, the rats were placed in the same device for 5 min, a period designated as the test period. The activity of the rats during the test was recorded with a video camera and the duration of immobility (defined as above), swimming (active horizontal movement, i.e. around or through the cylinder), or climbing (upward movement with the front paws pointing towards the cylinder wall) was measured over 5 min. The drug was administered orally 1 h before the test.

### 2.7 Locomotor activity in mice and rats

In order to confirm whether GW043 has an antidepressant activity, we used spontaneous activities in mice to determine whether GW043 affects the central system. In mice, 60 ICR mice were randomly allocation to six therapy groups (*n* = 10/group): control group, 10 mg/kg fluoxetine, 0.02 mg/kg GW043, 0.1 mg/kg GW043, 1.0 mg/kg GW043, and 10 mg/kg GW043. All mice received one administration (p.o.). After 30 min of gavage administration, each mouse was located in the corner of an open‐field chamber (50 × 50 × 50 cm) to adapt to 5 min. The numbers of crossings and rearing were recorded during the subsequent 5 min.

In rats, 36 SD rats were randomly allocation to 4 therapy groups (*n* = 9/group): control group, 0.02 mg/kg GW043, 0.1 mg/kg GW043, and 1.0 mg/kg GW043. All rats received one administration (p.o.). After 60 min of gavage administration, each rat was located in the corner of an open‐field chamber (100 × 100 × 50 cm) to adapt to 5 min. The numbers of crossings and rearing were recorded during the subsequent 5 min.

### 2.8 Chronic unpredictable mildstress (CUMS) procedure

**FIGURE 2:**
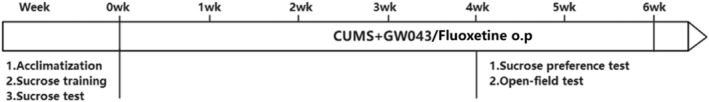
Flow chart of CUMS in Wistar rats.

To assess the antidepressant‐like effects of GW043, a CUMS model was established as described previously.^31^ After 1 week of habituation, 60 naïve rats underwent 48 h sucrose training and sucrose baseline testing. Rats were randomly divided into six groups according to their sucrose preference in the sucrose baseline test: control (non‐stressed), stress‐vehicle (distilled water), stress GW043 (0.1, 0.7 and 5.0 mg/kg), stress fluoxetine (10 mg/kg), and oral administration of excipients or drugs 1 h before stress (08:00–09:00). In addition to the control group, rats received multiple stressors: deprivation of food and water (24 h), overnight lighting, soiled cages (100 g wood chip bedding 200 mL water), low intensity strobe (100 times/min), forced swimming (5 min at 10°C), tail clamping (1 min), electric shock (10 s/time and three times), cage tilt at 45°C and restraint (2 h). The stressors were applied continuously and stochastically. Non‐stressed rats in the control group were given food and water free of charge, with the exception of a deprivation period of 14 h before each sucrose test. Alongside constant overnight lighting, all experimental animals were subjected to a consistent 12‐h cycle of light and darkness. After 4 weeks of stress, the sucrose preference test (day 29, rats on excipients or drugs for 28 d), the open field experiment (day 33, rats on excipients or drugs for 32 d) and the novelty inhibition feeding test (day 37, rats on excipients or drugs for 36 d) were performed without acute drug treatment. An outline of the design of the chronic unpredictable stress and behavior test is shown in Figure 2.

### 2.9 The open‐field test in the CUMS model

The apparatus used for the open‐field test was a region (100 × 100 × 50 cm) with a base divided equally into nine sectors. At 24 h after the last dose, the rats were placed in the centre of the arena and the number of crosses and rearing was recorded simultaneously over a period of 5 min.^32^

### 2.10 Sucrose preference test in CUMS model

Two bottles of 1% (w/v) sucrose solution were placed in each cage for the first 24 h. One of the bottles was replaced with pure water for the next 24 h. After 48 h of normal diet and water intake, water and food intake was prohibited for 14 h immediately following the baseline test 1 h. Baseline measurements were taken three times to obtain stable results (food and water are prohibited after normal intake each time). The sugar‐water preference index was calculated using the following formula.^33^ The sucrose preference [SP = sucrose intake × 100%/(sucrose intake + water intake)].

### 2.11 Golgi‐Cox staining of neuronal dendrites and dendritic spines

The use of FD Rapid Golgi Stain™ Kit for Golgi staining of neurons. Prepare impregnation solution (Solution A/B) by mixing equal volumes of Solutions A and B at least 24 h prior to use and leave unstirred. Deeply anesthetize experimental animal with IACUC‐approved methods before sacrificing. Remove animal brain (or postmortem human brain tissue) from the skull as quickly as possible. Be sure to handle carefully to avoid damaging or pressing the tissue. Rinse tissue quickly in double distilled or Milli‐Q water to remove blood from the surface. Immerse tissue in impregnation solution (Solution A/B) and store at room temperature for 3 weeks in the dark. Replace impregnation solution after the first 6 h of immersion or on the next day. Gently swirl (do not shake) the tissue container side to side for a few seconds at least twice a week during the impregnation period. Transfer tissue into Solution C and store at room temperature in the dark for at least 72 h (up to 1 week). Replace Solution C at least once after the first 24 h of immersion or on the next day. Freeze tissue for sectioning with a cryostat or sliding microtome. Cut 100‐ to 200‐μm sections using a cryostat (recommended) at −20°C to −23°C. Mount sections on gelatin‐coated microscope slides using Solution C. Prepare staining solution (Solution D/E), which consists of 1 part Solution D, 1 part Solution E, and 2 parts double distilled or Milli‐Q water. For example, mix the following solutions in the order listed: 10 mL Solution D, 10 mL Solution E, and 20 mL double‐distilled water. Rinse sections in double distilled or Milli‐Q water two times for 4 min each rinse. Replace distilled water after each use. Place sections in the staining solution for 10 min. Rinse sections in double distilled or Milli‐Q water two times for 4 min each rinse. Replace distilled water after each use. Dehydrate sections in sequential rinses of 50%, 75%, and 95% ethanol, 4 min each rinse. Dehydrate sections in 100% ethanol four times for 4 min each rinse. Clear sections in xylene three times for 4 min each rinse. Coverslip sections using mounting medium. Image sections using an upright bright‐field microscope. Store Golgi‐stained sections at room temperature protected from light.

Under a 100× objective, clear cell dendrites were randomly searched in the CA3‐CA1 region of the hippocampus. Three to five secondary parietal and basal dendrites having at least one branch point were selected for measurement. Count the visible vertebrae along the branching segments and the data are expressed as vertebrae number/10 μm.

### 2.12 5‐Bromodeoxyuridine (BrdU) staining

The experimental procedure is as described previously.^34^ BrdU powder was dissolved in 0.9% saline and injected intraperitoneally with 50 mg/kg at 12 h intervals 3 days before execution. 1 d after the last BrdU injection, animals were anesthetized with an overdose of sodium pentobarbital and then perfused intracardially with 0.9% saline and 4% paraformaldehyde. All brain tissue was then immersed in 30% sucrose solution and serially frozen at 40 μm after 48 h at a thickness of −3.30 to −4.52 mm. 10 serial sections were collected on slides and stored at −20°C.

Transfer slices from the cryoprotection solution to0.1 M PBS until it reaches room temperature. Rinse three times for 10 min each with 0.1 M PBS. Perform antigen retrieval if required. Incubate for 20 min in 2 N HCl at 37°C. Rinsein 0.1 M borate buffer (8.5 pH) for 10 min. Rinse three times for 10 min each with ice‐cold 0.1 M PBS. Incubate for 2 h at room temperature with blocking solution. Incubate with 1:250 anti‐BrdU primary antibody (mouse host) in PBS+ (add 3% (3 mL) of normal horse serum and 0.3% (300 μL) of Triton X‐100 to 0.1 M PBS (pH 7.4)) overnight at 4°C. On day 2, rinse three times for 10 min each with 0.1 MPBS. Incubate with 1:250 fluorochrome‐conjugated secondary antibody (anti‐mouse) in PBS+ for 2–4 hat room temperature. Rinse three times for 10 min each with 0.1 M PBS. Carefully mount slices on gelatinized slides using as oft brush, air dry overnight at room temperature, or mount immediately with an appropriate mounting medium. Counterstain, add permanent mounting medium and place coverslips. Store at 4°C for up to 6 months.

The number of BrdU‐positive cells in the dorsal hippocampus was counted under a 40 × objective (8 sections per animal). In each section, the outline of the granular cell layer (GCL) and the subgranular zone (SGZ; a two‐cell wide area just below the GCL) were sketched out first under the 10× objective. After switching to a 40× lens, the software randomly selected approximately 30 counting frames within the outline, with the BrdU‐positive cells in each counting frame being counted by the observer. Following the calculation of all 8 parts of an animal, the software estimated the total number of cells in that animal.

### 2.13 Western blotting analysis

Tissues from hippocampal and prefrontal regions were cut into small pieces, placed in EP tubes, homogenized, and lysis solution (Beyotime, Nanjing, China, main components 20 mM Tris (pH 7.5), 150 mM NaCl, 1% Triton X‐100, 1 mM PMSF, sodium pyrophosphate, sodium β‐glycophosphate, ethylene disodium EDTA, Na3VO4, and leucine), and the supernatant was collected by centrifugation at 12,000 rpm for 10 min. Protein concentrations were determined using the BCA Protein Kit. Based on the protein quantification results, a corresponding volume of the total protein sample was added to 5 × protein loading buffer. Following electrophoresis, the polymeric protein bands separated from the gel were shifted onto polyvinylidene difluoride (PVDF) membranes by transfer electrophoresis. The membranes were incubated with primary and secondary antibodies in turn, and then subjected to radiographic autoradiography. The intensities of the spots were analyzed by Image Pro plus 6.0. Data were obtained from three rats per group. The mean values for each group were calculated and plotted.

### 2.14 Statistical analysis

Analysis was performed using GraphPad Prism 8 (GraphPad Software Inc., San Diego, CA, USA). Differences between groups were identified by one‐way ANOVA and LSD's test. Comparisons between the two groups were made using the t‐test. All the data are presented as the mean ± standard error. *p < 0.05 was regarded as a significant difference.

## 3 RESULTS

### 3.1 GW043 has no significant affinity for depression‐related receptors in the CNS (non‐NMDAR)

**FIGURE 3:**
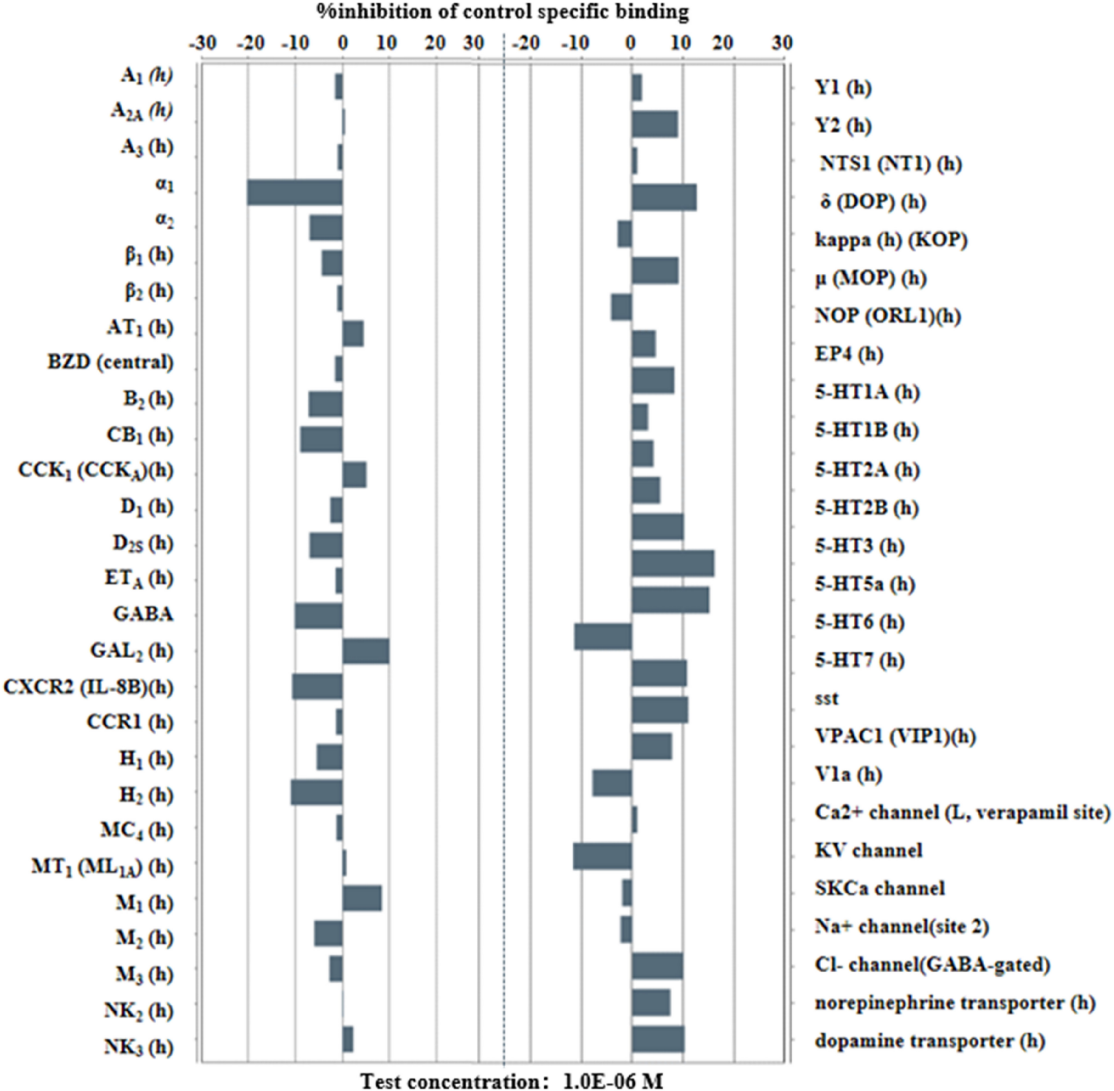
Screening of GW043 targets by receptor‐ligand binding assay. Affinity of GW043 for depression‐related receptors in the CNS (non‐NMDAR). Inhibitory or agonistic activity below 50% was considered as no significant affinity. Detailed experimental methods and data are presented in Data S1.

As shown in Figure 3 below, GW043 1μM has no significant affinity for 55 depression‐related receptors in the CNS other than NMDAR.

### 3.2 GW043 partially activates NMDAR and promotes LTP in hippocampus

**FIGURE 4:**
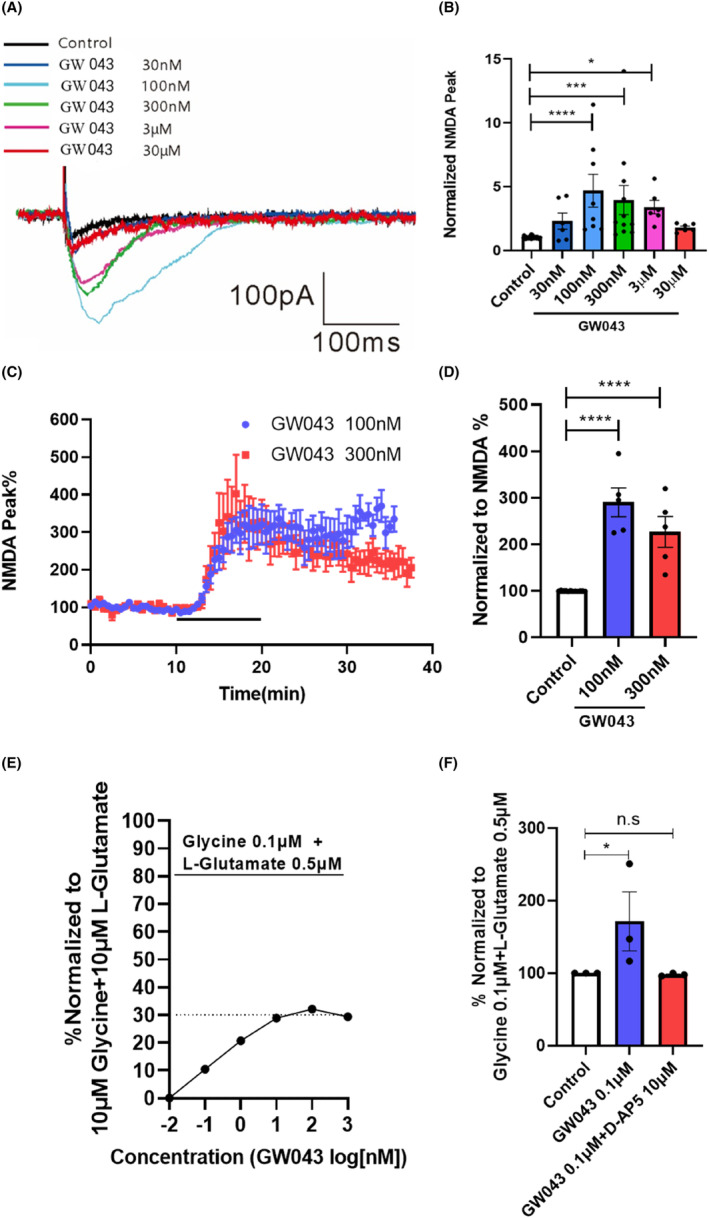
Electrophysiological techniques to explore the action characteristics of GW043 on NMDAR. (A) Peak curves of NMDAR currents in the hippocampal CA1 region of C57BL/6 mice induced by different concentrations of GW043, (B) the current amplitude values of NMDAR in the hippocampal CA1 region of C57BL/6 mice after adding different concentrations of GW043 (*n* = 6–11, cell), (C) peak curves of NMDAR currents in CA3‐CA1 region of SD rat hippocampus induced by GW043, (D) percentage of peak NMDAR currents in CA3‐CA1 region of SD rat hippocampus induced by GW043 after normalization (*n* = 5, cell), (E) NMDAR currents induced by GW043 as a percentage of it induced by the saturating agonist glycine + L‐glutamate in African clawed toad oocytes (*n* = 3, cell), (F) effect of NMDAR blocker D‐AP5 on the NMDAR currents induced by GW043 (*n* = 3, cell). Data are expressed as Means ± SEM. *p < 0.05, ***p < 0.001, and ****p < 0.0001.

Following the establishment of a stable baseline, we proceeded to experimentally assess the impact of GW043 on NMDAR currents. As illustrated in Figure 4A,B, we observed the direct impact of GW043 (100 nM, 300 nM and 3 μM) on the NMDAR located in the vertebral cells of the CA1 region of the hippocampus in the C57BL/6 mice (*F*_5,69_ = 7.767, *p* < 0.0001). Meanwhile, GW043 (100 and 300 nM) increased the current amplitude in vertebrate cell NMDA receptors in the CA3‐CA1 region of the hippocampus of SD rats (*F*_2,19_ = 34.20, *p* < 0.0001), as Figure 4C,D. In addition, for oocytes expressing rNR1/NR2B, different concentrations of GW043 induced channel currents, meanwhile the amplitude of maximum activation was 32.13% of the full agonist (10 μM Glycine +10 μM L‐Glutamate) (Figure 4E). And the application of D‐AP5 (10 μM) entirely negated the influence of GW043 (0.1 μM) on the amplitude of NMDAR currents as observed in African clawed toad oocyte (*F*_2,6_ = 4.130, *p* < 0.05), as Figure 4F.

**FIGURE 5:**
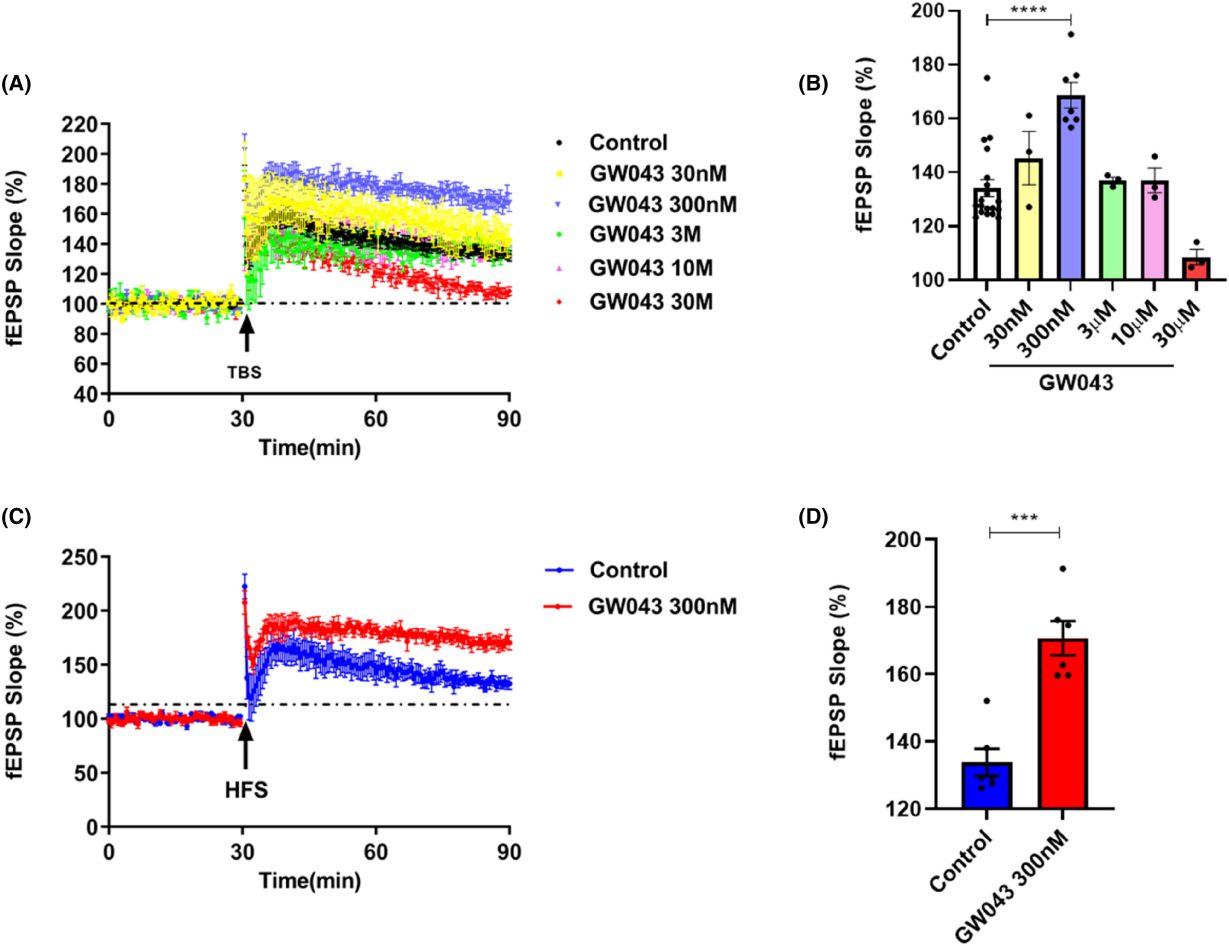
Effect of GW043 on TBS‐ and HFS‐induced LTP in the hippocampal CA3‐CA1 region of C57BL/6 mice, (A) and (B) effect of different concentrations of GW043 on TBS‐induced LTP (*n* = 3–7, mice), (C) and (D) effect of 300 nM of GW043 on HFS‐induced LTP (*n* = 5, mice). Data are expressed as mean ± SEM. ***p < 0.001 and ****p < 0.0001.

As shown in Figure 5, GW043 at a 300 nM concentration notably heightened the fEPSP slope amplitude in the CA3‐CA1 region of the hippocampus in C57BL/6 mice slices upon being induced by both theta burst stimulation (TBS) (*F*_5,32_ = 11.93, *p* < 0.0001) and high‐frequency stimulation (HFS) (*t* = 5.676, *p* < 0.0001).

### 3.3 The immobility time is shortened in FST after an acute administration of GW043

**FIGURE 6:**
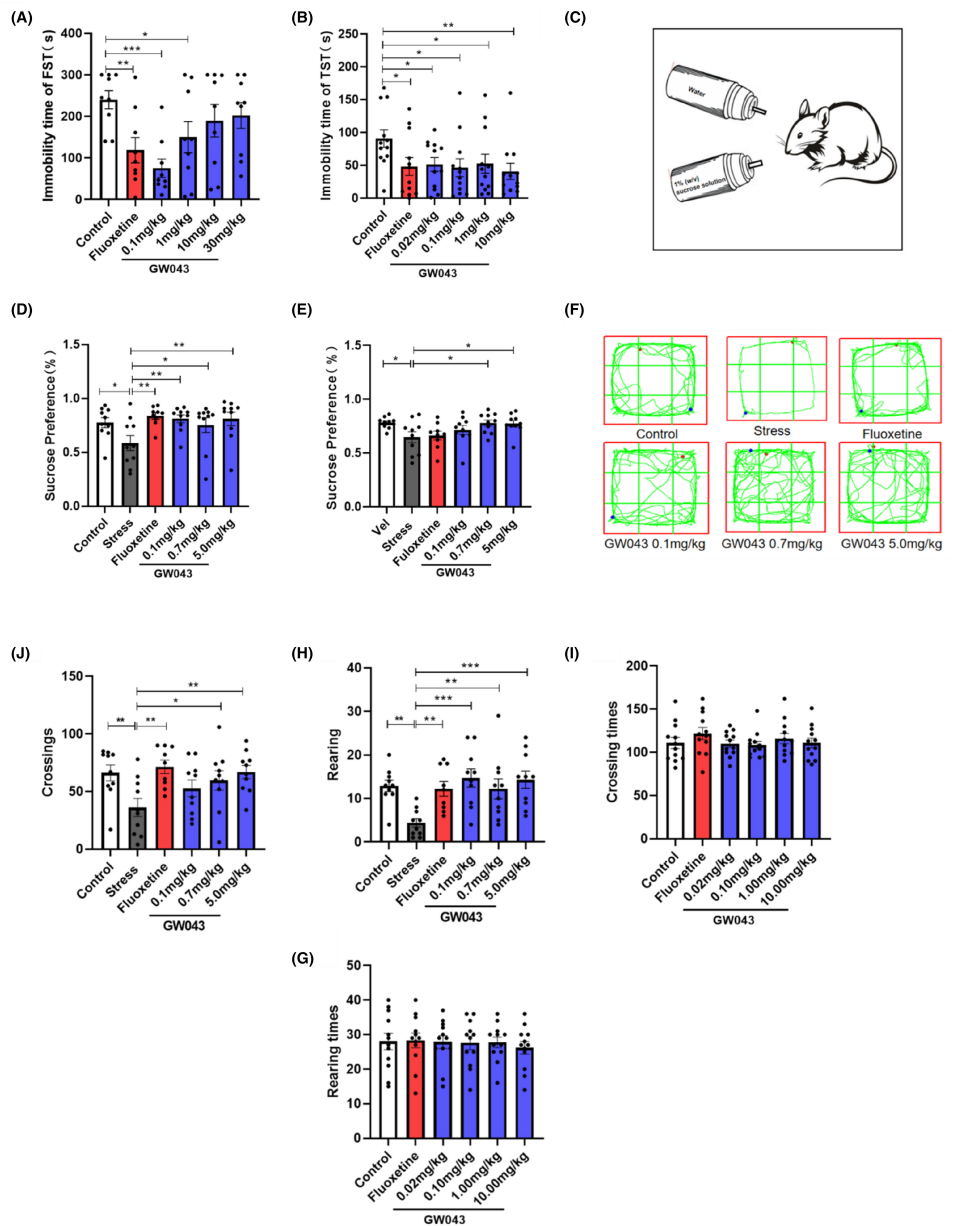
Behavioral tests were performed after acute dosing and CUMS. (A) Effect of fluoxetine (10 mg/kg) and GW043 (0.1, 0.7, and 5.0 mg/kg) on the immobility time in FST of SD rats (*n* = 10, rats), (B) effect of fluoxetine (10 mg/kg) and GW043 (0.1, 0.7, and 5.0 mg/kg) on the immobility time in TST of ICR mice (*n* = 12, mice), (C) schematic diagram of sucrose preference test, (D) effects of fluoxetine (10 mg/kg) and GW043 (0.1, 0.7, and 5.0 mg/kg) administration on sucrose preference in CUMS rats at week 4 (*n* = 9–10, rats), (E) effects of fluoxetine (10 mg/kg) and GW043 (0.1, 0.7, and 5.0 mg/kg) administration on sucrose preference in CUMS rats at week 2 (*n* = 9–10, rats), (F) trajectory of CUMS rats in the open‐field tests, as well as the number of (J) crossings and (H) raring (*n* = 9–10, rats). (I, G) Effect of GW043 on spontaneous activity in normal mice (*n* = 12, mice). Data are expressed as the means ± SEM. *p < 0.05, **p < 0.01, and ***p < 0.001.

The administration of fluoxetine at a dosage of 10 mg/kg resulted in a significant decrease in the immobility time of rats in the FST compared to the control group (Figure 6A), suggesting that the behavioral test was conducted under standard and reliable conditions. A one‐way analysis of variance (ANOVA) revealed significant differences among the experimental groups of rats in the FST. Specifically, the immobility time of rats in the GW043 dose groups of 0.1 and 1.0 mg/kg was significantly reduced (*F*_5,48_ = 3.711, *p* < 0.05).

### 3.4 The immobility time is shortened in TST after an acute administration of GW043

Figure 6B illustrates that GW043, when administered at doses of 0.02, 0.10, 1.00, and 10.00 mg/kg, resulted in a significant decrease in the duration of immobility in the TST in mice. Similarly, the administration of fluoxetine (10 mg/kg) led to a notable reduction in the immobility time of the mice in the TST (*F*_5,66_ = 1.916, *p* < 0.05).

### 3.5 GW043 increase the sucrose preference in CUMS rats

An illustration of the effect of GW043 on sucrose preference in CUMS rats is shown in Figure 6D. Four‐weeks after stress, the sucrose preference of rats in the stress‐vehicle group was statistically lower than that of the control group, indicating that developing CUMS model successfully. And as a positive control, fluoxetine (10 mg/kg) significantly increased sucrose preference in CUMS rats, demonstrating the predictive validity of CUMS model. Chronic administration of GW043 primarily affected sucrose preference in CUMS rats. Further post hoc analyses revealed that sucrose preference returned to normal levels in the GW043 (0.1, 0.7 and 5.0 mg/kg, p.o. administration for 28 d) groups of rats (*F*_5,53_ = 2.780, *p* < 0.05).

### 3.6 GW043 reverses the reduction in locomotor activity in CUMS rats

The effect of GW043 on the open‐field behavior of CUMS rats is shown in Figure 6. The number of crossings and rearing (Figure 6J,H) were significantly lower in the stress‐vehicle group of rats after 4 weeks of exposure to CUMS than in the control group (crossings: stress vs control, *p* < 0.01, rearing: stress vs control, *p* < 0.01), indicating that setting CUMS model up successfully. As a positive control, fluoxetine (10 mg/kg) markedly increased the number of crossings and rearing in chronically stressed rats, demonstrating the predictive validity of the CUMS model. Further retrospective analysis showed that GW043 (p.o. administration for 28 d) at 0.1, 0.7 and 5.0 mg/kg reversed the inhibition of locomotor activity in CUMS rats, and the number of crossings (*F*_5,53_ = 3.286, *p* < 0.05) and rearing (*F*_5,53_ = 4.464, *p* < 0.05) were significantly increased in the GW043‐.treated groups compared to the stress‐vehicle group.

### 3.7 Locomotor activity in mice and rats

Table 1 describes the role of GW43 on spontaneous activity in mice and rats. In mice, GW043 (0.02, 0.1, 1.0, or 10 mg/kg) given by gavage had no effect on the number of crossings (*F*_5,66_ = 0.7739, *p* = 0.5720) and rearing (*F*_5,66_ = 0.1424, *p* = 0.9816) in the spontaneous activity test. In rats, GW043 (0.02, 0.1 or 1.0 mg/kg) given by gavage also had no effect on the number of crossings (*F*_3,32_ = 0.1380, *p* = 0.9365) and rearing (*F*_3,32_ = 0.06748, *p* = 0.9768).

**TABLE 1:** Effects of GW43 on locomotor activity in mice and rats. Data are presented as mean ± SEM (*n* = 9–10/group).

Species	Dose of GW043 (mg/kg)	Locomotor activity	
		Crossing number	Rearing number
Mice	Control	110.9 ± 6.537	28.00 ± 2.396
	Fluoxetine	121.8 ± 7.047	28.33 ± 2.154
	0.02	109.9 ± 4.126	27.83 ± 1.878
	0.10	108.3 ± 4.384	27.58 ± 1.960
	1.00	116.0 ± 5.866	27.75 ± 1.558
	10.00	110.9 ± 5.621	26.17 ± 1.808
Rats	Control	51.11 ± 6.783	9.000 ± 1.394
	0.02	46.56 ± 5.480	8.667 ± 1.374
	0.1	45.67 ± 7.205	9.444 ± 1.717
	1.0	50.33 ± 9.173	9.667 ± 2.261

### 3.8 GW043 promotes neuronal regeneration and synaptic spine density

**FIGURE 7:**
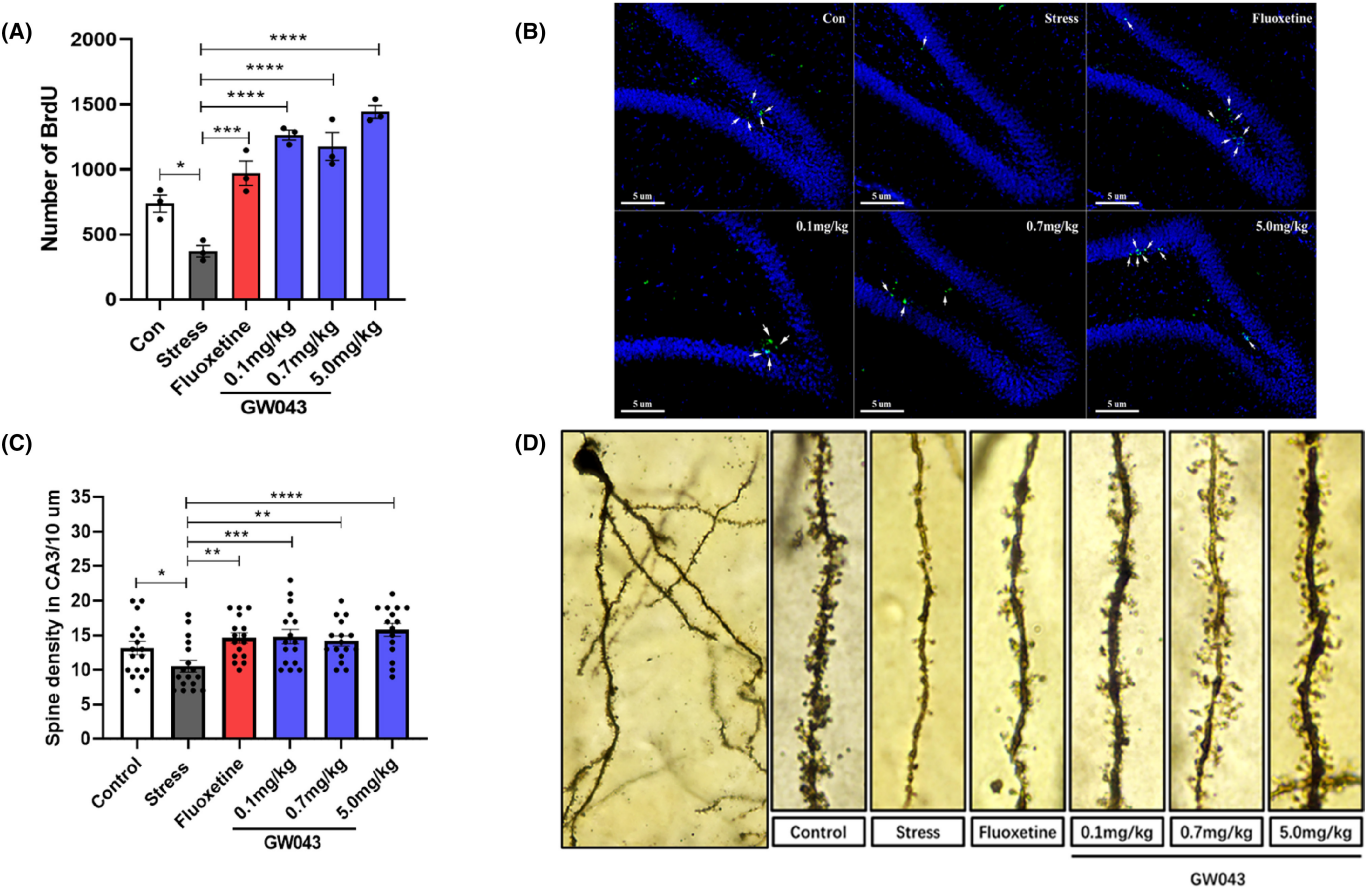
Observations of neuronal regeneration by BrdU injection and synaptic morphology by Golgi staining after 4 weeks of CUMS. (A, B) Effects of 4‐week administration of fluoxetine (10 mg/kg) and GW043 (0.1, 0.7, and 5.0 mg/kg) on neuronal regeneration in CUMS rats (*n* = 3, rats). (C, D) Effects of 4‐week administration of fluoxetine (10 mg/kg) and GW043 (0.1 mg/kg, 0.7 mg/kg, and 5.0 mg/kg) on synaptic spine density in CUMS rats (*n* = 15–17 branch, 3 rats). Data are expressed as the mean ± SEM. *p < 0.05, **p < 0.01, ***p < 0.001, and ****p < 0.0001.

BrdU‐positive cells in the DG of hippocampus were observed as shown in Figure 7A,B. Cell proliferation was significantly reduced in the hippocampus's DG region of rats in the stress‐vehicle group, and the number of BrdU positive cells was reduced compared to the control group (*p* < 0.05). Fluoxetine treatment reversed CUMS‐induced BrdU‐positive cytopenia. The findings suggest that GW043 treatment protects the hippocampus from the negative effects of CUMS, which was a significant difference in the number of BrdU‐positive cells in the DG (*F*_5,12_ = 30.30, *p* < 0.0001).

As shown in Figure 7C,D, a comparison of the spine densities on secondary dendrites in the CA3 region of the hippocampus between the stressed and non‐stressed groups revealed a significant difference (*p* < 0.05). As a positive control, fluoxetine (10 mg/kg) markedly increased the spine densities on secondary dendrites in the CA3 region of the hippocampus, demonstrating the predictive validity of the CUMS model. GW043 also increased the density of spines compared to the stress‐vehicle group. Long‐term gavage administration of GW043 (0.1, 0.7 and 5.0 mg/kg) increased dendritic spine density in the CA3 region of the hippocampus in the group of rats (*F*_5,91_ = 4.328, *p* < 0.05).

### 3.9 GW043 upregulates BDNF expression and mTOR phosphorylation in the hippocampus and prefrontal lobe of CUMS rats

**FIGURE 8:**
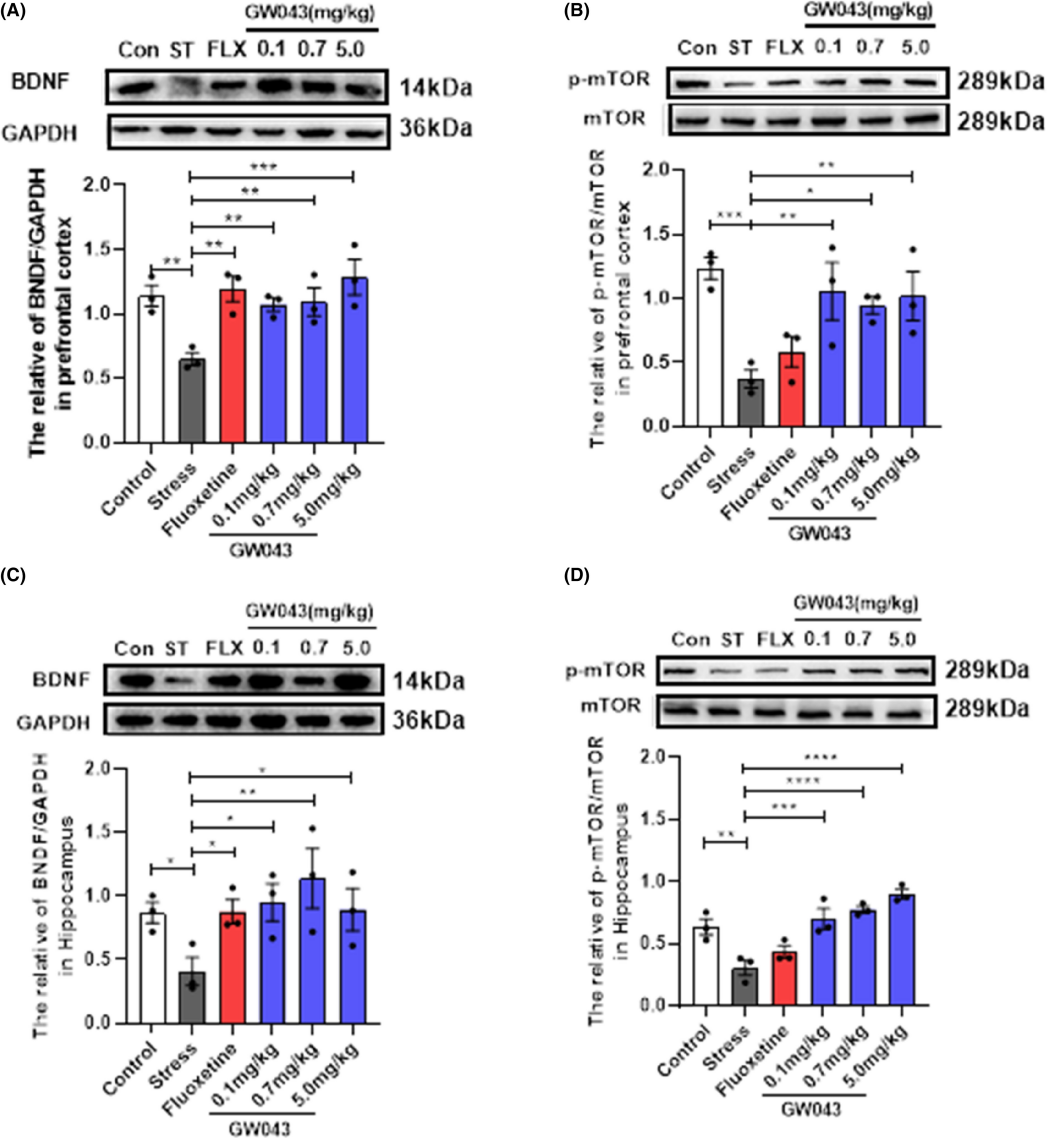
Western blot detection of changes in the levels of BDNF and mTOR phosphorylation in the prefrontal lobe and hippocampus of CUMS rats. (A, B) Effect of GW043 on the levels of BDNF and mTOR phosphorylation in the prefrontal lobe of CUMS rats (*n* = 3, rats). (C, D) Effect of GW043 on the levels of BDNF and mTOR phosphorylation in the hippocampus of CUMS rats (*n* = 3, rats). In the figure, Con is control, ST is stress, and FLX is fluoxetine, data are expressed as means ± SEM. *p < 0.05, **p < 0.01, ***p < 0.001, and ****p < 0.0001.

The dysregulation of BDNF and p‐mTOR molecules within the BNDF‐mTOR signaling pathway in the hippocampus and prefrontal lobes is believed to play a crucial role in the pathophysiology of depression. Consequently, we conducted a comparative analysis of the alterations in BNDF and p‐mTOR content in rats subjected to CUMS. Our findings, as depicted in Figure 8C,D, demonstrate a significant impact of CUMS exposure on the concentration of BDNF and p‐mTOR in the hippocampus. Stress‐induced decreases BDNF (*F*_5,12_ = 2.638, *p* < 0.05) and p‐mTOR (*F*_5,12_ = 14.92, *p* < 0.01) levels are markedly reversible in rats treated with GW043 (0.1, 0.7, 5.0 mg/kg). Fluoxetine (10 mg/kg) administration elevates BDNF in CUMS rats, however, no visible effect on p‐mTOR.

In the prefrontal lobe (Figure 8A, B), stress invited a reduction in the levels of BNDF (*F*_5,12_ = 5.614, *p* < 0.01) and p‐mTOR (*F*_5,12_ = 5.342, *p* < 0.05), with both GW043 and fluoxetine have similar effects on BDNF and p‐mTOR as in the hippocampus.

## 4 DISCUSSION

GW043 is a newly developed compound possessing a unique molecular structure that has been specifically designed and synthesized with the aim of selectively targeting the NMDAR. Previous studies have established that when administered orally at a dosage of 3 mg/kg, GW043 exhibits an approximate oral bioavailability of 60%, with translocation across the blood–brain barrier occurring at an estimated rate of 3% in rodent models. Our investigative results demonstrate that GW043 has shown significant efficacy toward NMDAR *in vitro*, enhancing LTP in *ex vivo* animal brain tissue. Within animal models, GW043 has displayed a potent ability to attenuate behaviors associated with depression, thus highlighting its potential use as an antidepressant. Pursuant to biochemical analysis, it can be inferred that this mechanism of action is primarily based on the BDNF‐mTOR signaling pathway.

Through employing patch‐clamp recording, a dose–response curve was engineered to assess the impact of GW043 on the NMDAR currents' amplitude in the hippocampus vertebral cells of C57BL/6 mice. The data revealed an increase in the NMDAR‐EPSC amplitude following the administration of low concentrations of GW043 (100 nM–3 μM) (Figure 4A,B). This effect was reliably replicated in vertebral cells of SD rats at concentrations of 100 nM and 300 nM (Figure 3C,D). This allowed us to confirm the agonistic activity of GW043 on NMDAR. To validate the functionality of GW043, we utilized the expression of NMDARs incorporating rNR1/NR2B subunits in Xenopus oocytes. Notably, we observed that the maximum agonistic effect of GW043 amounted to 32.13% of that exerted by the full agonist (10 μM glycine +10 μM L‐glutamate) (Figure 4E), while the agonism of GW043 was blocked by the NMDAR antagonist D‐AP5 (Figure 4F). The observed features presented by this evidence bear resemblance to those documented previously for NMDAR partial agonists rapastinel,^26^, ^35^ leading us to postulate that GW043 is plausibly a partial agonist of NMDAR.

To affirm the selectivity of GW043 for NMDA receptors, we screened 55 other depression‐associated receptors in the central nervous system, with NMDA receptors excluded. The experimental results revealed that GW043 demonstrated no notable affinity for any of the measured receptors, thereby providing further evidence of its specific targeting toward NMDA receptors (Figure 3).

Animal behavioral experiments are widely recognized as crucial for investigating human pathogenesis and evaluating the effectiveness of new drugs. Classical behavioral models, such as the TST, FST, and CUMS, are widely utilized as behavioral assessment methods to evaluate the effectiveness of antidepressants, owing to their several advantages, which include ease of use, consistent results, and straightforward measurement.^36^, ^37^ Initially, the acute antidepressant effects of GW043 were examined in ICR mice using FST and TST, and our findings unequivocally established significant antidepressant effects at doses ranging from 0.02–10 mg/kg (Figure 6B, Figure S1). To ascertain the antidepressant effects across different species, we performed the FST on SD rats. Remarkably, GW043 displayed a therapeutic window of 0.02–1 mg/kg (Figure 6A, Figure S1). The efficacy of GW043 was convincingly demonstrated in the widely accepted CUMS model. Through 4 weeks of simultaneous oral administration, GW043 effectively reinstated the sucrose preference and spontaneous activity of CUMS rats to a level comparable to normal rats, affirming its notable antidepressant effect (Figure 6C–H). Noteworthy is the fact that GW043 exhibited an earlier onset of action in terms of sucrose preference rate when compared to the conventional antidepressant fluoxetine (Figure 6E).

In order to rule out the potential influence of central nervous system stimulants, we evaluated the spontaneous activity in normal ICR mice and SD rats after administering GW043. Gratifyingly, the findings indicated the absence of any central excitatory effect caused by GW043 (Table 1). Hence, the aforementioned studies provide evidence of the rapid (compared to fluoxetine) and substantial antidepressant effect of GW043.

Ketamine has been reported to preferentially block NMDAR on intermediate neuronal subpopulations leading to glutamate bursts followed by activation of AMPAR which stimulates BDNF release that in turn activates TrkB receptors and downstream signaling pathways such as MEK–ERK, P13K–Akt, and mTORC1. Moreover, it has been evidenced that ketamine effectively inhibits extrasynaptic NMDAR, leading to the blockade of eEF2 synthesis. Consequently, this inhibition induces BDNF synthesis and enhances protein synthesis, along with an increase in AMPAR cycling.^38^, ^39^, ^40^ These effects ultimately promote synapse formation and lead to an augmentation in the density of dendritic spines.^38^ In contrast, rapastinel acts as a partial agonist at the NMDAR glycine site depending on L‐type voltage‐dependent calcium channels (VDCCs) and AMPA receptors, increasing cellular Ca^2+^ influx thereby leading to BDNF release and activation of the BDNF/TrkB signaling pathway.^41^, ^42^ However, it should be emphasized that the activation of mTORC1 is indispensable for the behavioral and synaptic implications of rapastinel. The antidepressant effects of rapastinel in several behavioral models including the FST, novelty suppressed feeding test (NSFT), and female sniff urine test (FUST) in rats were completely blocked by rapamycin, a selective mTORC1 inhibitor.^43^ In view of the well‐documented fact that mTORC1 and BDNF signaling are also necessary for ketamine action, rapastinel shares the same downstream pathway as ketamine, showing convergent effects leading to a rapid and long‐lasting antidepressant response. Therefore, we explored the effects of GW043 on BDNF and mTOR phosphorylation in prefrontal and hippocampal regions in CUMS rats by western blot. The results showed that GW043 reversed the downregulation of BDNF and mTOR phosphorylation by CUMS in the prefrontal lobe, a phenomenon replicated in the hippocampus (Figure 8, Figure S4). These findings indicate that GW043 exerts its antidepressant effects by regulating BDNF and mTOR signaling pathways through NMDAR activation.

LTP, a form of synaptic plasticity that depends on specific events, is prevalent in the majority of synapses within the nervous system. Serving as a significant indicator of synaptic information storage processes, LTP is widely recognized as a key candidate for the cellular substrates of learning and memory,^44^, ^45^, ^46^ and the principal type of LTP is thought to be dependent on the activation of high calcium‐permeable ionotropic NMDAR.^47^ Among the several models correlated to synaptic plasticity, rapastinel treatment showed persistent behavioral effects, with significant increases in LTP induced by maximal stimulation in physiological studies, lasting at least 2 weeks with single doses of rapastinel and repeated administration for at least 8 weeks.^27^, ^48^ Thus rapastinel is reasonable to assume that its long‐lasting antidepressant‐like effects involve NMDAR‐mediated processes analogous to LTP. Similarly, our data reveal that different concentrations of GW043‐induced LTP when administered in the CA3‐CA1 region of C57 mice, in the presence of TBS induction (Figure 5). Also, with HFS guidance, GW043 at 300 nM achieves the same results. Then, we observed that GW043 had a significant effect on the synaptic morphology of the hippocampus of CUMS rats, that is, the density of synaptic spines was denser in GW043‐treated rats than in the stress group (Figure 7C,D and Figure S2). This explains the rapid onset and long‐lasting effects of GW043 compared to fluoxetine in treating depression‐like behavior in animals and provides empirical evidence for the hypothesis that ketamine and rapastinel. At the same time, electrophysiological and mechanistic studies have given us hints that GW043 has the potential for rapid onset of action in an orally administered form.

Multiple pieces of evidence support the notion that the effects of antidepressants could be linked to their capacity to promote hippocampal neurogenesis in both animals and non‐human primates.^49^, ^50^, ^51^ Moreover, neurogenic disorders are thought to be strongly associated with depression and could be induced by CUMS at the same time.^52^ Thus, we investigated the impact of GW043 on the proliferation of hippocampal cells through BrdU immunohistochemical staining. The findings indicate that prolonged administration of GW043 led to an enhancement in cell proliferation within the dentate gyrus region of the hippocampus, in comparison to the CUMS stress group (Figure 7A,B and Figure S3). This result further corroborates the antidepressant effect of GW043 at the cellular level.

In summary, our investigation reveals that GW043, acting as a partial agonist of NMDAR, exhibits remarkable antidepressant effects in rodent models. The underlying mechanisms involved in these effects are proposed to include the modulation of synaptic plasticity through the LTP and BDNF‐mTOR pathways, leading to an upsurge in the generation of new neurons and subsequent antidepressant actions.

## 5 CONCLUSION

Our findings provide evidence that GW043 functions as a partial agonist of NMDAR, exerting substantial antidepressant effects across various models including FST, TST, and CUMS. Importantly, GW043 does not manifest any central nervous system excitatory or inhibitory effects. Mechanistic investigations further indicate that GW043 modulates synaptic plasticity via the LTP and BDNF‐mTOR pathways, facilitating an increase in the proliferation of newborn neurons, thereby eliciting its antidepressant effects.

## AUTHOR CONTRIBUTIONS

Participated in research design: Murezati Tiliwaerde and Zengliang Jin. Conducted experiments: Murezati Tiliwaerde and Nana Gao. Performed data analysis: Murezati Tiliwaerde, Nana Gao, and Yaqi Yang. Contributed to the writing of the manuscript: all authors.

## CONFLICT OF INTEREST STATEMENT

The authors declare that there are no conflicts of interest.

## Supporting information


Figure S1.

Figure S2.

Figure S4.

Data S1.

## Data Availability

The data that support the findings of this study are available from the corresponding author upon reasonable request.
