# Supplementary material for: A novel NMDA receptor modulator: the antidepressant effect and mechanism of GW043
Source: CNS Neurosci Ther. 2024 Feb 8;30(2):e14598. doi: 10.1111/cns.14598 (PMC10853642; doi:10.1111/cns.14598)
Supplement: Supplementary file 1 — Figure S1. [file CNS-30-e14598-s002.docx]

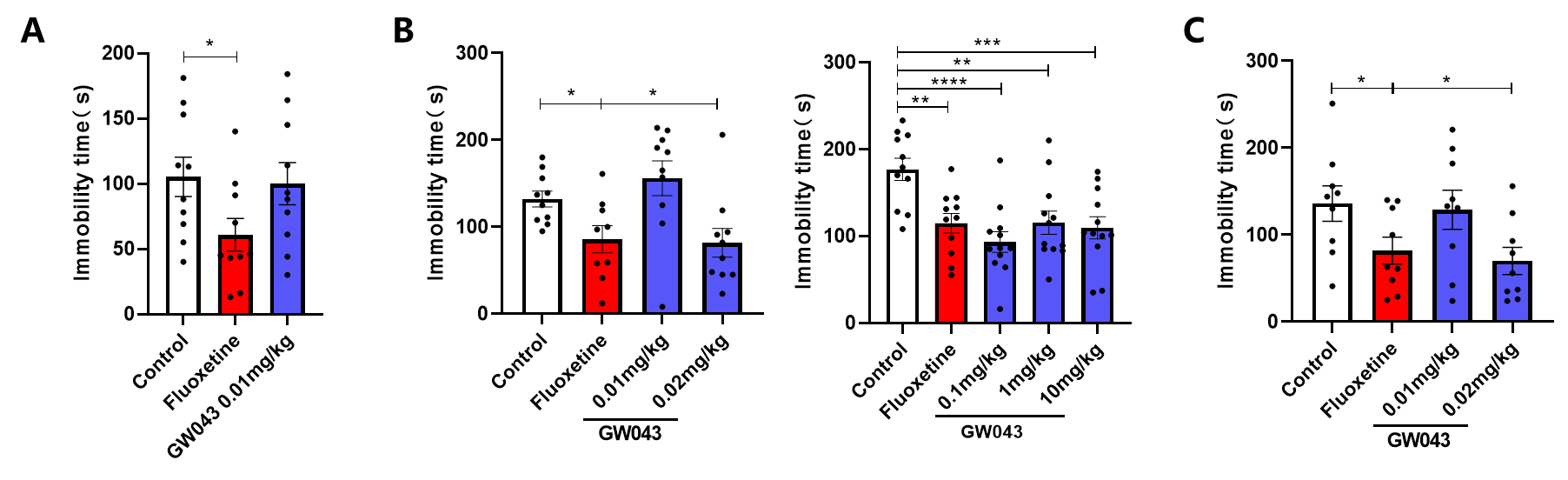


Supplementary figure1: Additional experiment on quantifying quantity-effect relationships in GW043. (A) Effect of fluoxetine (10 mg/kg) and GW043 (0.01 mg/kg) on the immobility time in TST of mice (n=10), (B) effect of fluoxetine (10 mg/kg) and GW043 (0.01-10 mg/kg) on the immobility time in FST of mice (n=9-12), (C) Effect of fluoxetine (10 mg/kg) and GW043 (0.01 mg/kg and 0.02 mg/kg) on the immobility time in FST of SD rats (n=9). Data are expressed as the Means±SEM, *p < 0.05.
