# Supplementary material for: A novel NMDA receptor modulator: the antidepressant effect and mechanism of GW043
Source: CNS Neurosci Ther. 2024 Feb 8;30(2):e14598. doi: 10.1111/cns.14598 (PMC10853642; doi:10.1111/cns.14598)
Supplement: Supplementary file 2 — Figure S2. [file CNS-30-e14598-s003.docx]

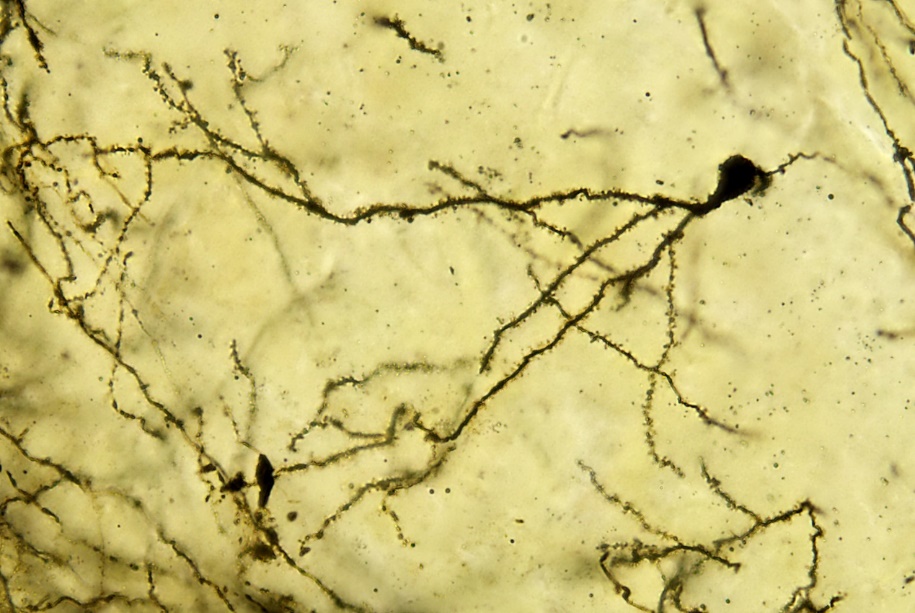


**B**

**A**


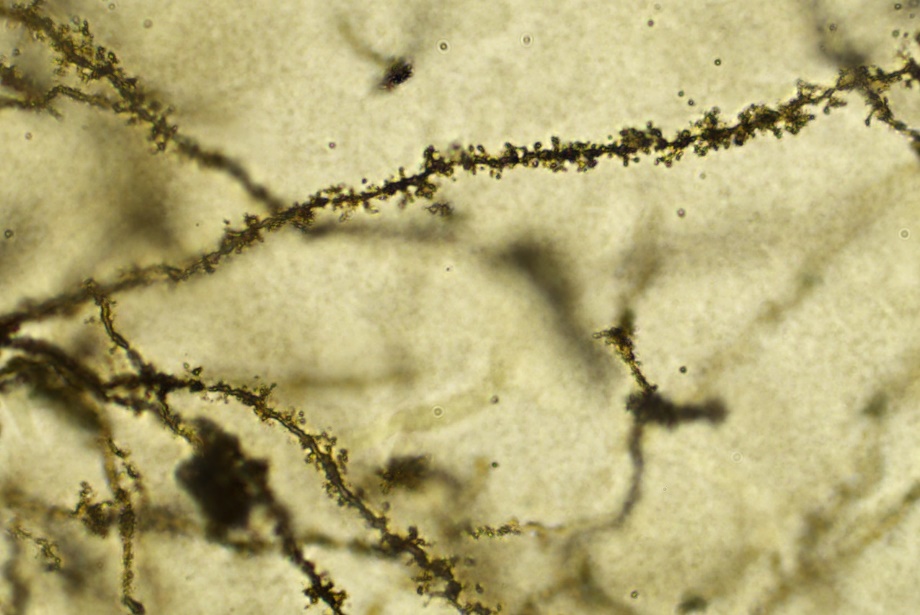


**
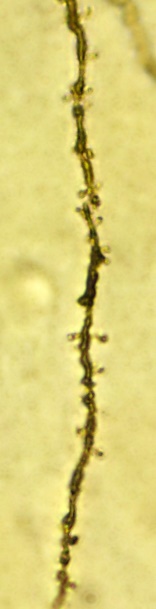

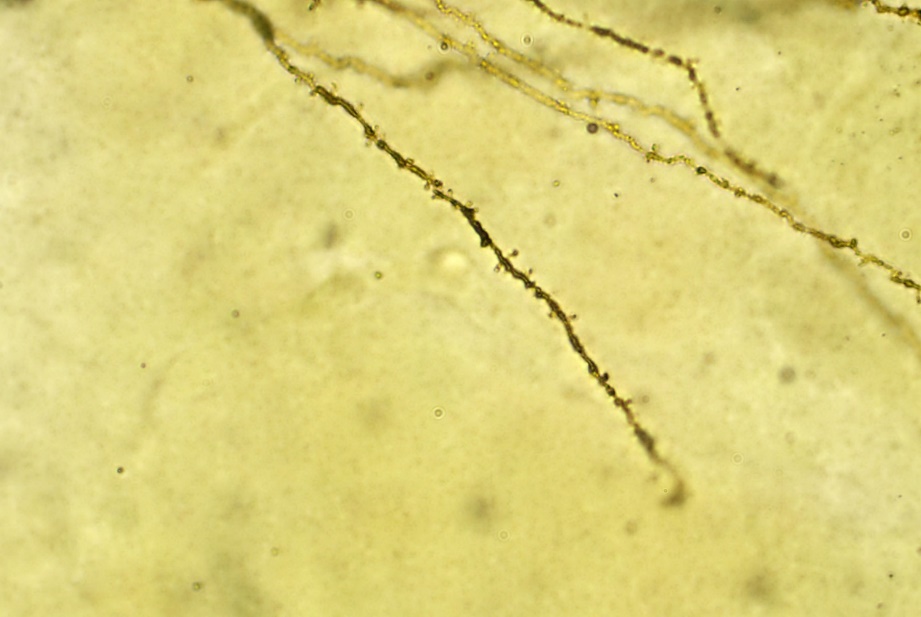
**
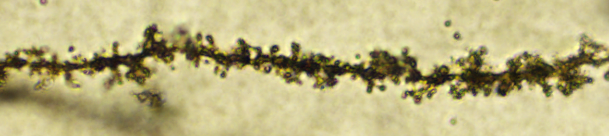


**C**

**
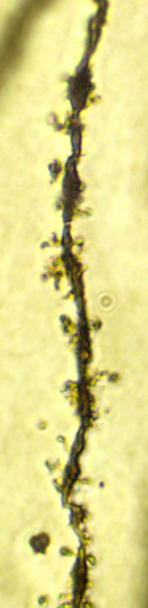

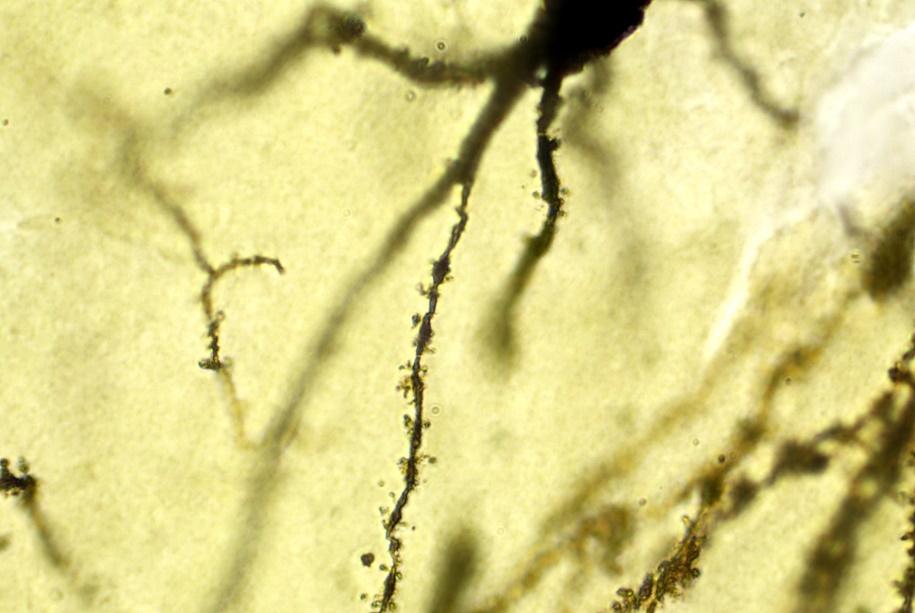
**

**D**

**
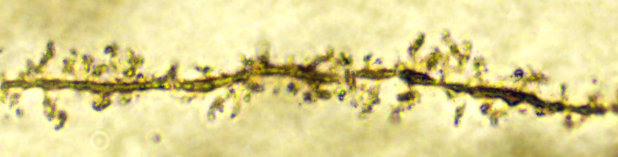

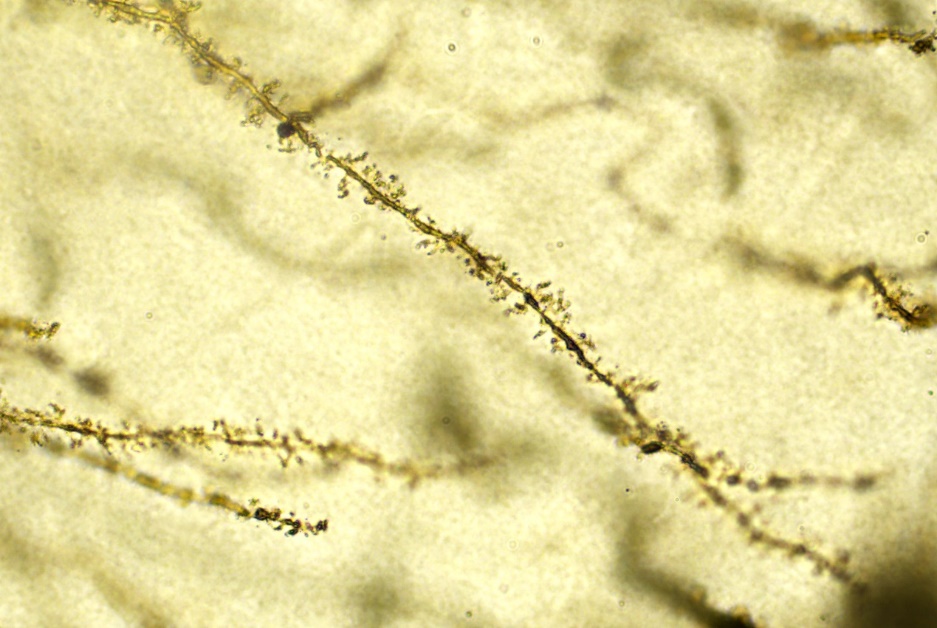

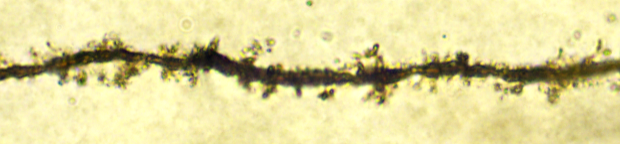

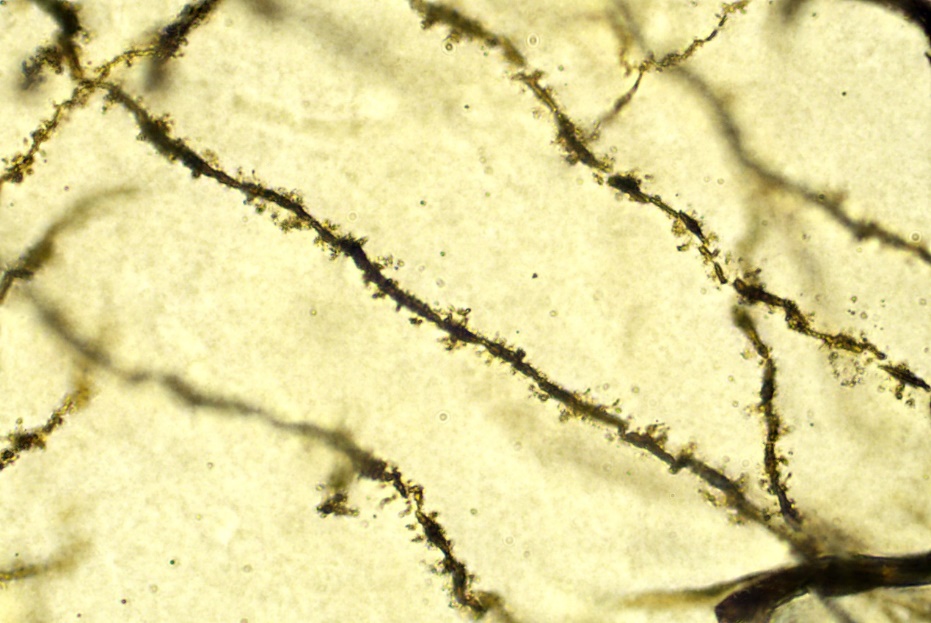
**

**F**

**E**

**
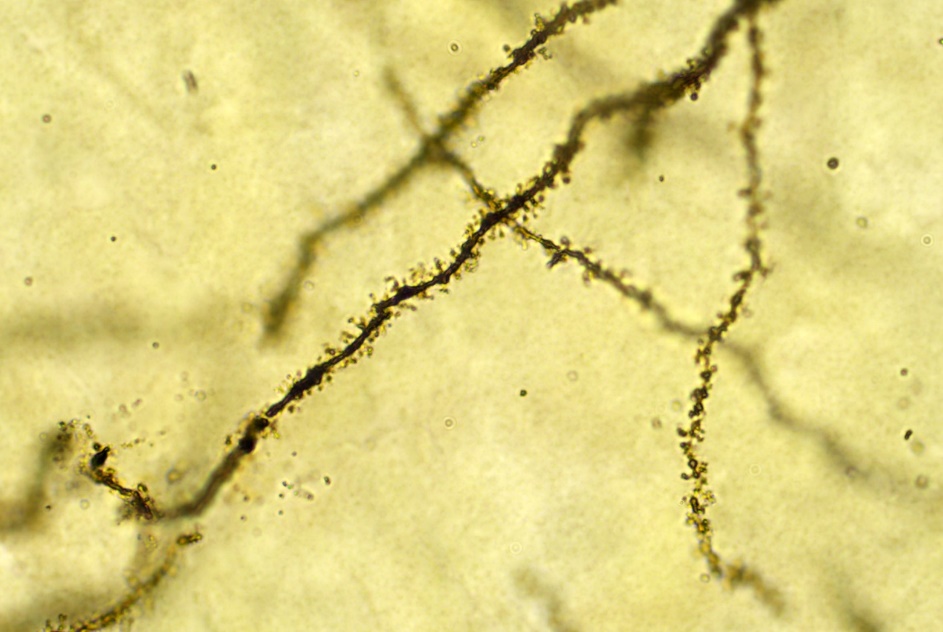
**

**G**

**
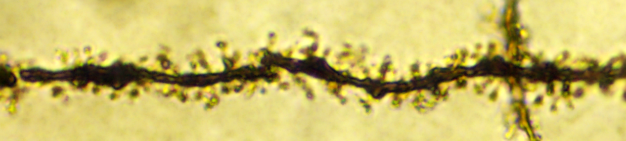
**

Supplementary figure2: Original Golgi staining image (Figure7D full unedited image). (A) Unedited diagram of a neuron under a microscope. (B) Control group full unedited image. (C) Stress group full unedited image. (D) Fluoxetine group full unedited image. (E) GW043 0.1mg/kg group full unedited image. (F) GW043 0.7mg/kg group full unedited image. (G) GW043 5.0mg/kg group full unedited image.

**
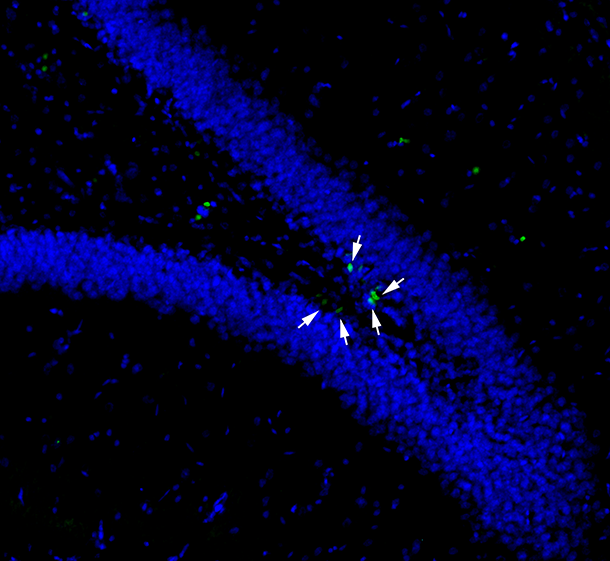
**

**B**

**A**

**
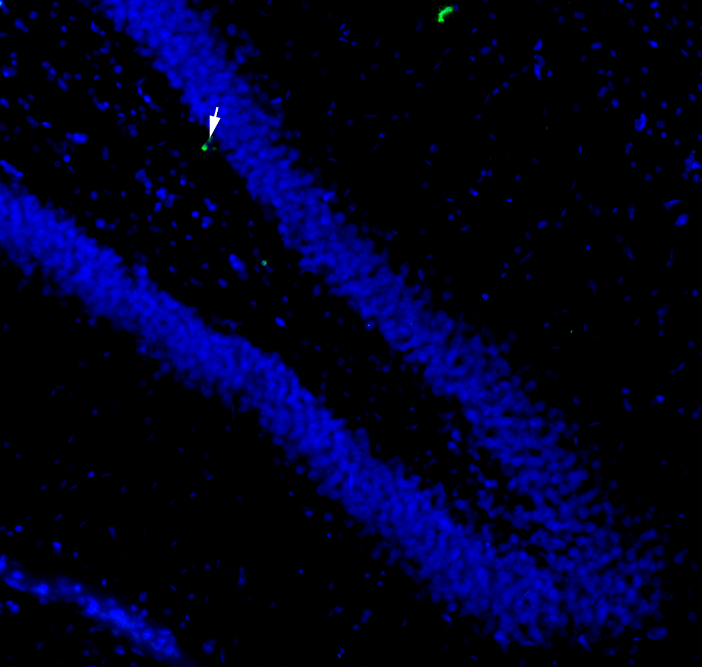
**

**C**

**
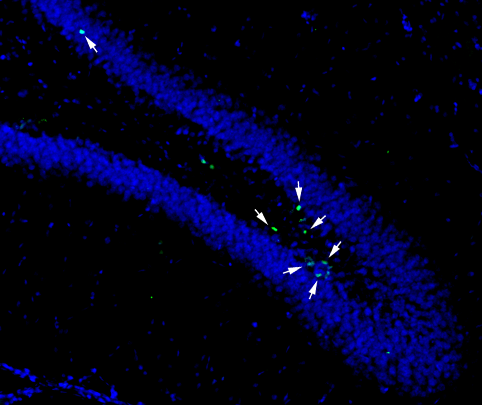
**

**
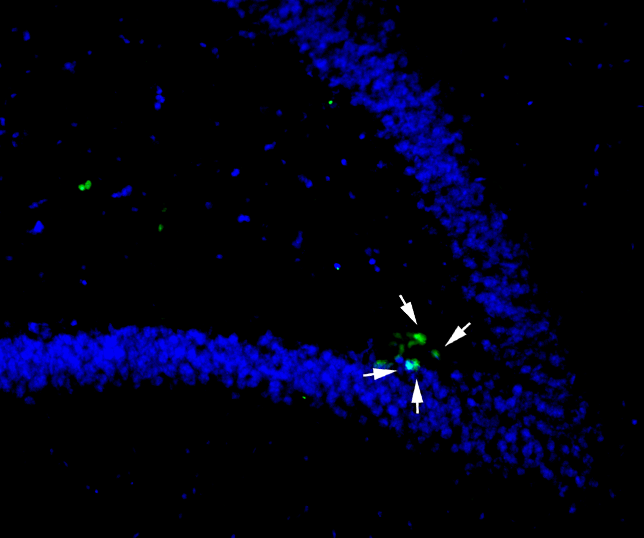
**

**E**

**D**

**
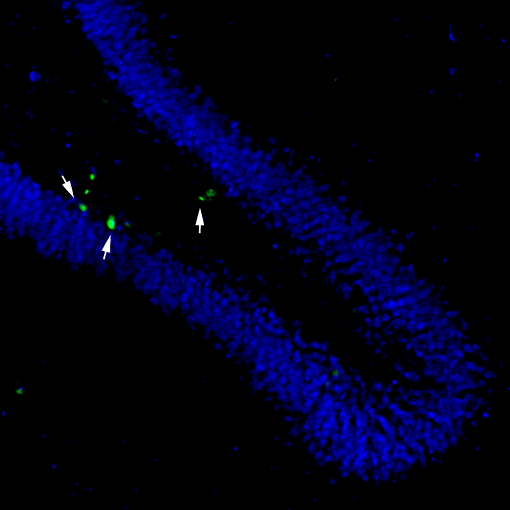
**
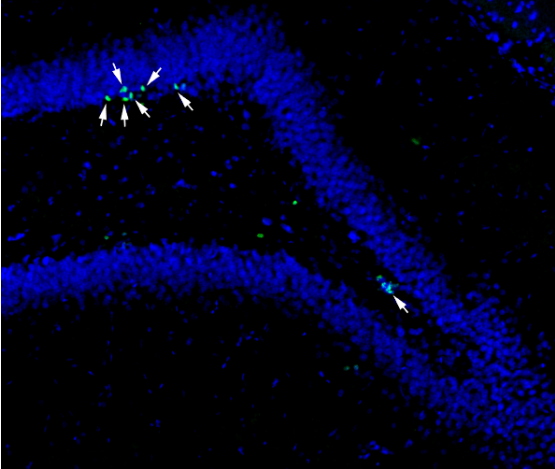


**F**

Supplementary figure3: Original BrdU staining image (Figure7B full unedited image). (A) Control group full unedited image. (B) Stress group full unedited image. (C) Fluoxetine group full unedited image. (D) GW043 0.1mg/kg group full unedited image. (E) GW043 0.7mg/kg group full unedited image. (F) GW043 5.0mg/kg group full unedited image.
