# Supplementary material for: A novel NMDA receptor modulator: the antidepressant effect and mechanism of GW043
Source: CNS Neurosci Ther. 2024 Feb 8;30(2):e14598. doi: 10.1111/cns.14598 (PMC10853642; doi:10.1111/cns.14598)
Supplement: Supplementary file 3 — Figure S4. [file CNS-30-e14598-s004.docx]

**
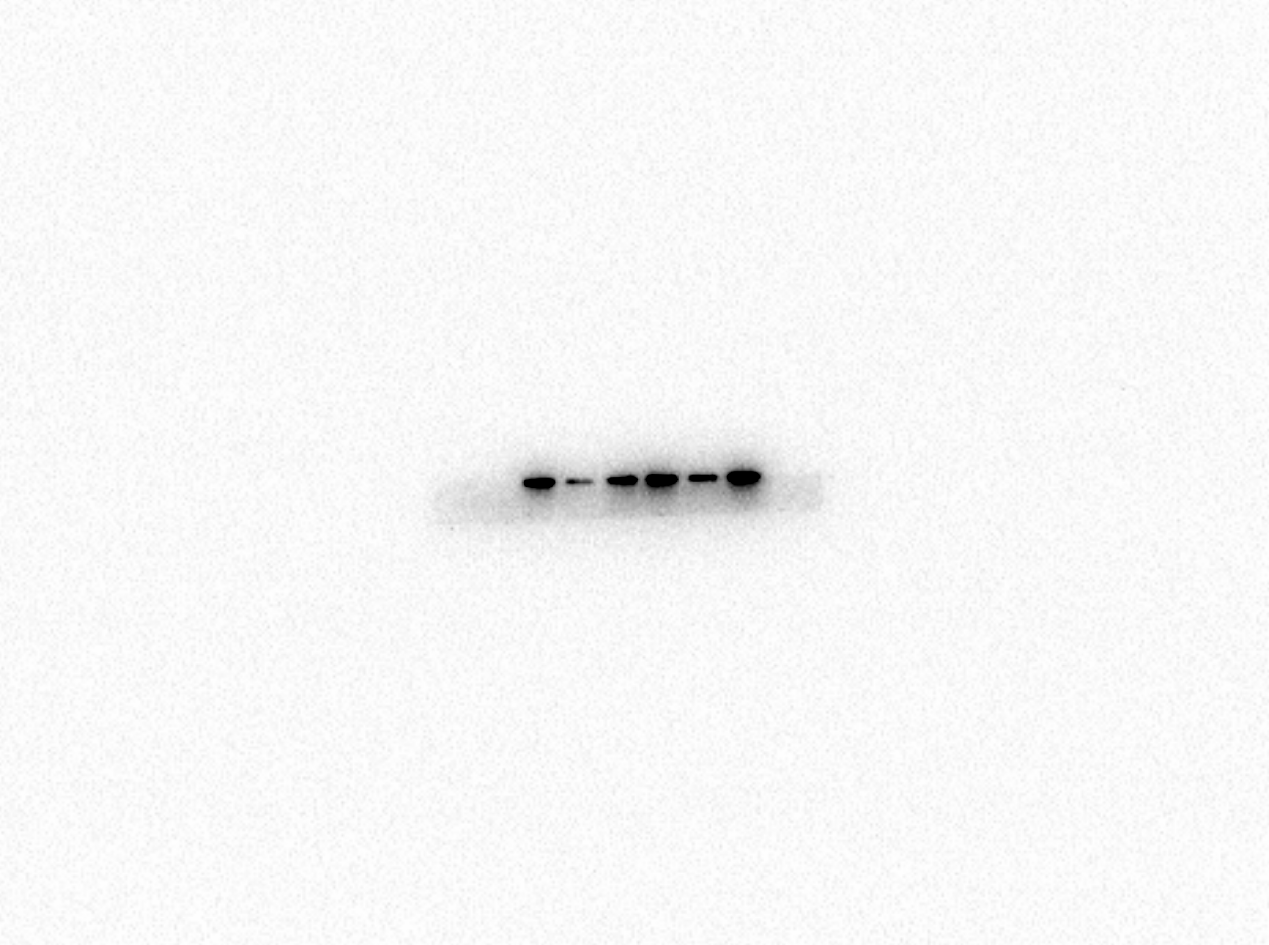
**

**B**

**A**

**
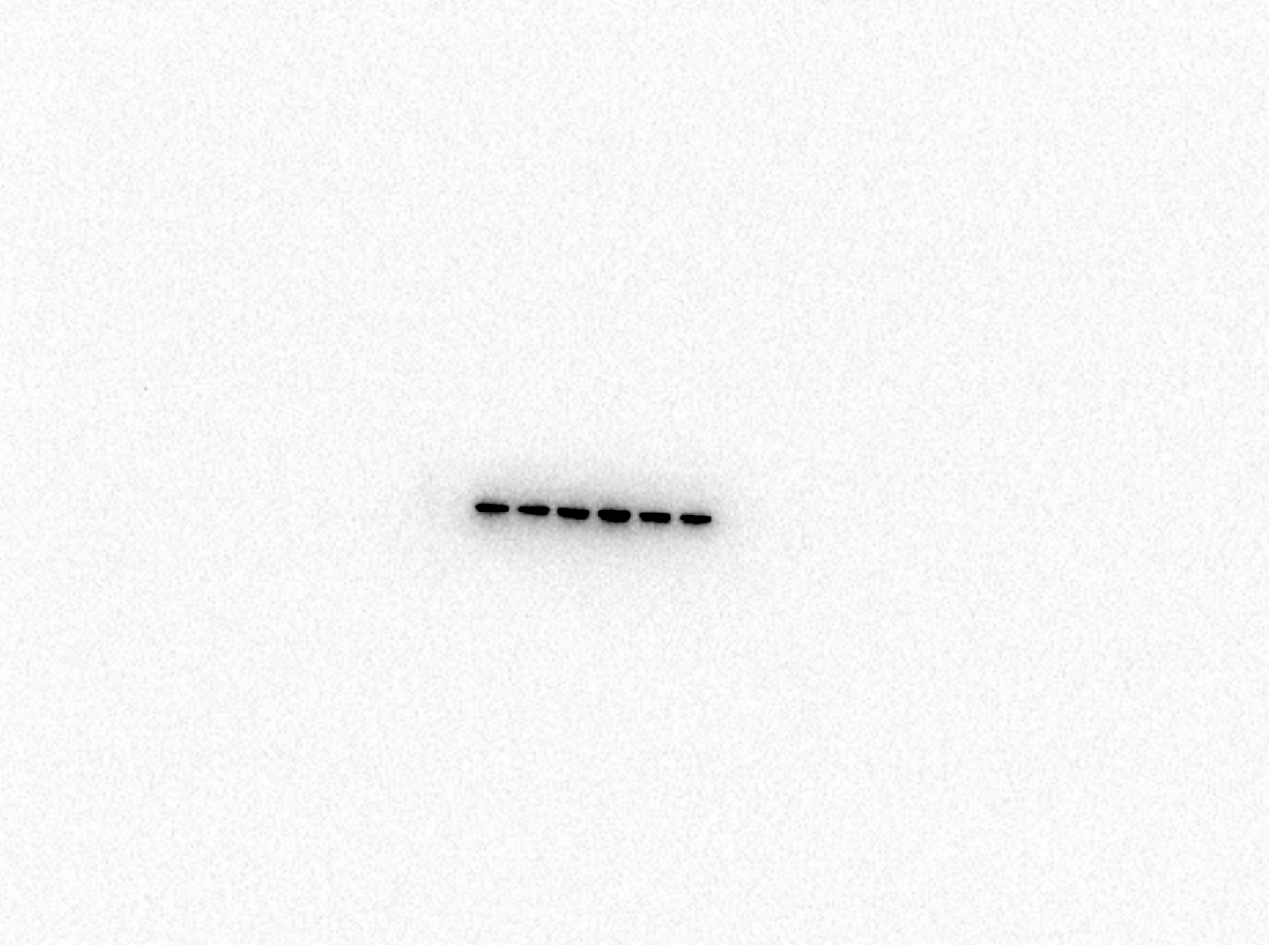
**

**C**

**

**

**D**





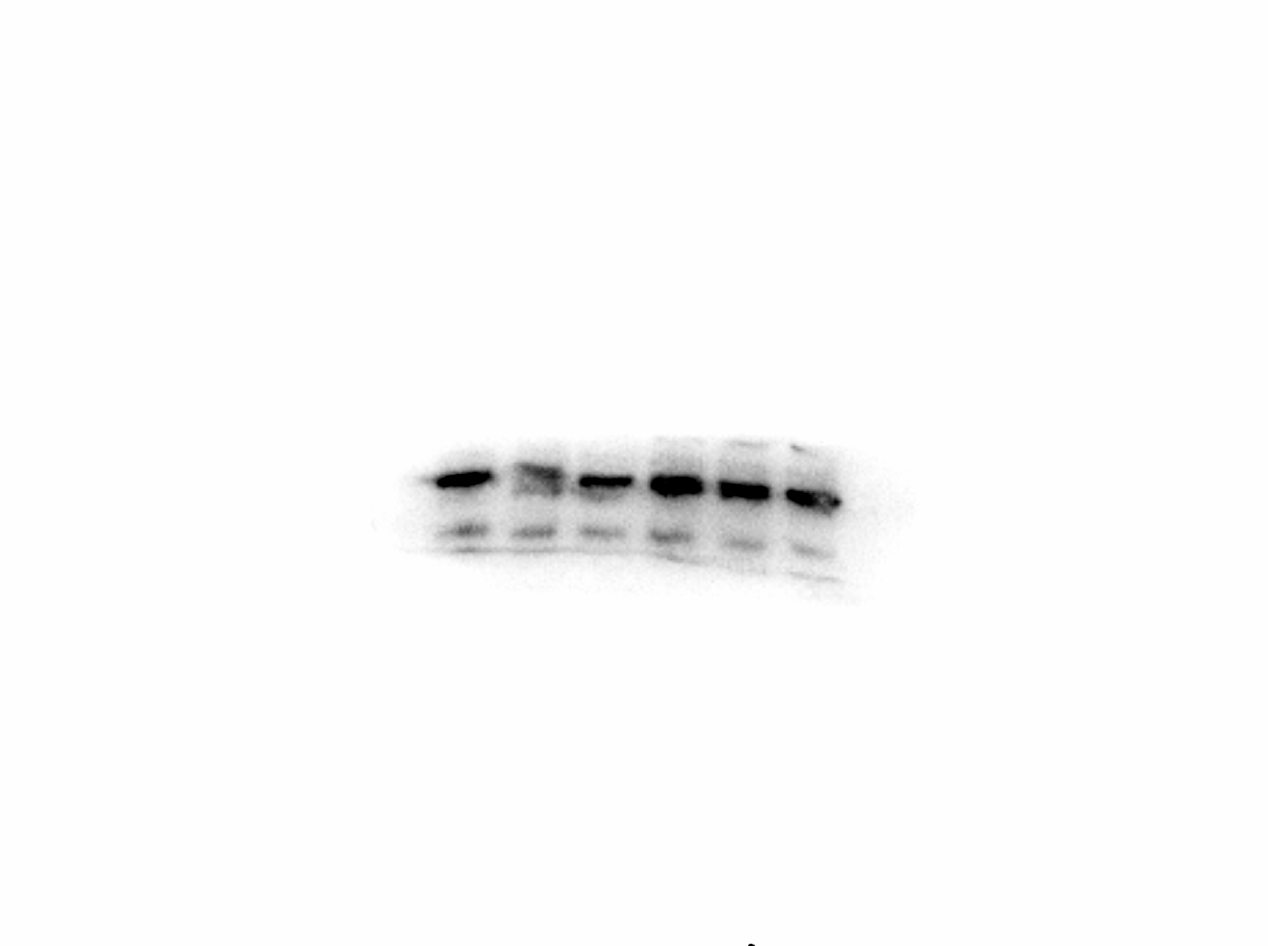


**E**


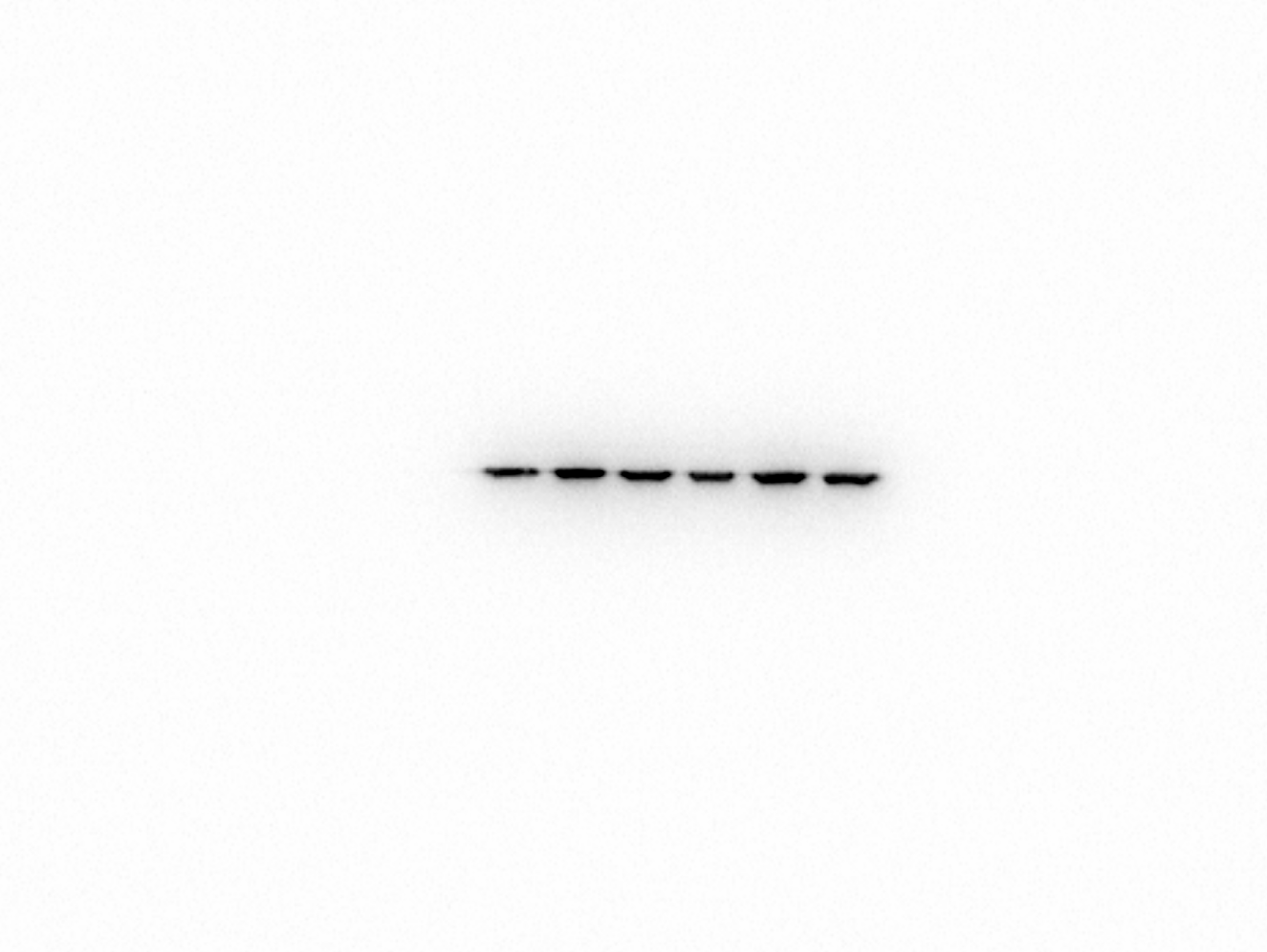


**G**

**F**


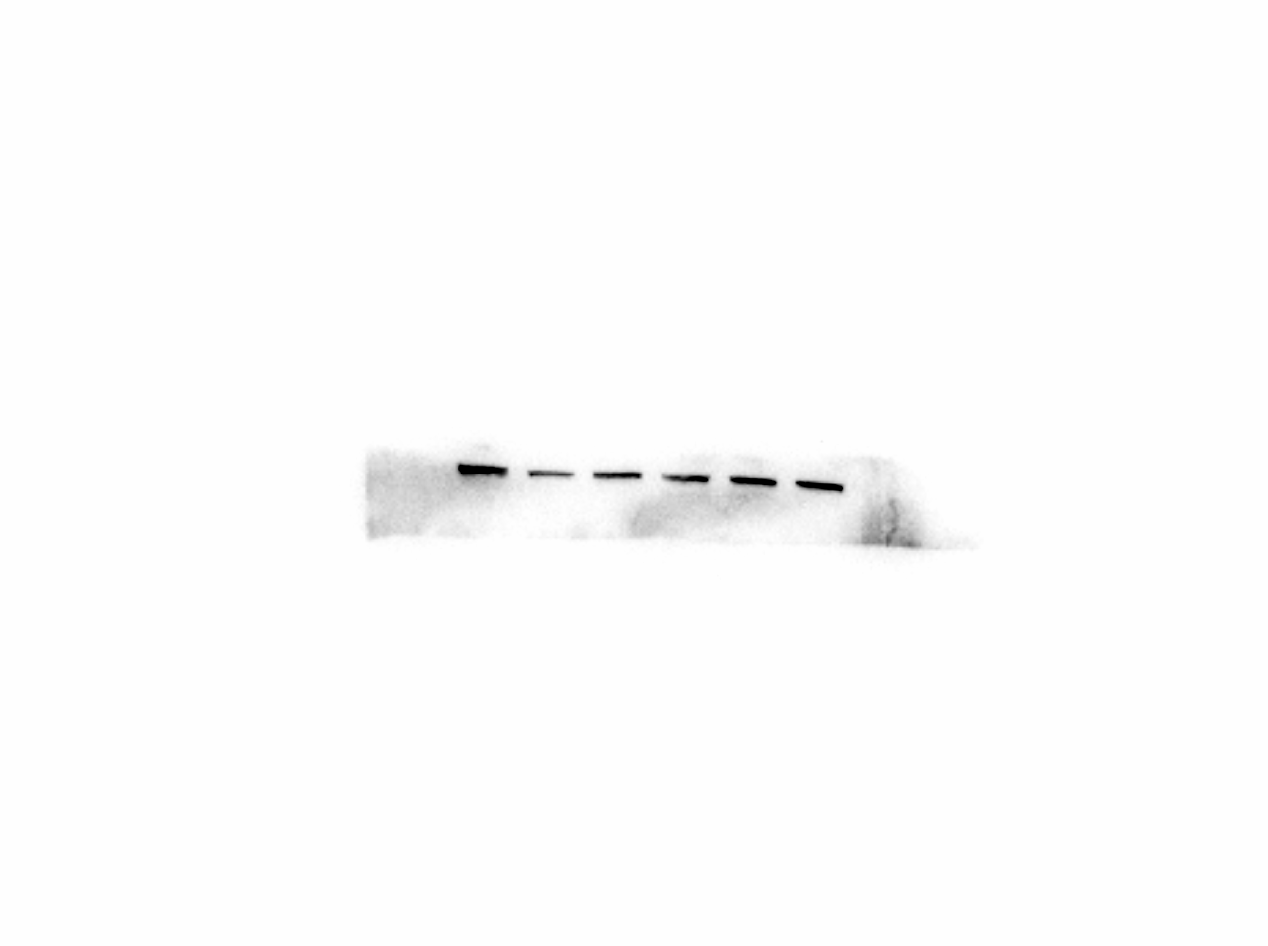


**H**


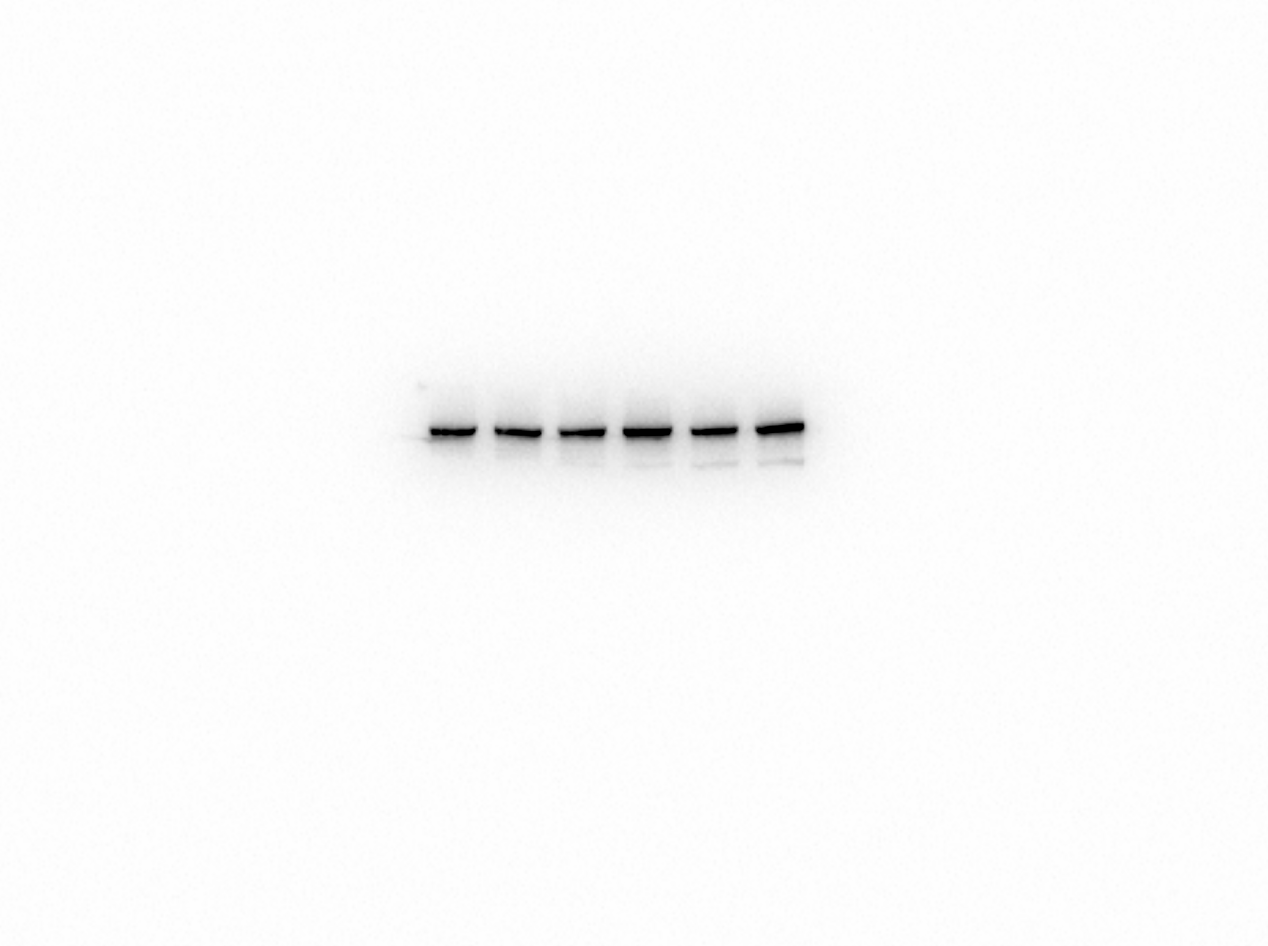


Supplementary figure4: Original image of western blot (Figure8 full unedited image). (A) Hippocampus BDNF (Full unedited blot for Figure 8C). (B) Hippocampus GAPDH (Full unedited blot for Figure 8C). (C) Hippocampus p-mTOR (Full unedited blot for Figure 8D). (D) Hippocampus mTOR (Full unedited blot for Figure 8D). (E) Prefrontal cortex BDNF (Full unedited blot for Figure 8A). (F) Prefrontal cortex GAPDH (Full unedited blot for Figure 8A). (G) Prefrontal cortex p-mTOR (Full unedited blot for Figure 8B). (H) Prefrontal cortex mTOR (Full unedited blot for Figure 8B).
