# Supplementary material for: A novel NMDA receptor modulator: the antidepressant effect and mechanism of GW043
Source: CNS Neurosci Ther. 2024 Feb 8;30(2):e14598. doi: 10.1111/cns.14598 (PMC10853642; doi:10.1111/cns.14598)
Supplement: Supplementary file 4 — Data S1. [file CNS-30-e14598-s001.docx]

1. **Methods**
2. **Tail suspension test (TST) in mice**

30 ICR mice were randomly divided into 3 groups (n=10/group): control group, 10.00 mg/kg fluoxetine, 0.01 mg/kg GW043. The drug was administered via gavage 30 minutes prior to the initiation of the experiment. One week after adaptive feeding, the TST was conducted: the hanging tail box was 25×25×35 cm, and using the tape to hang the mouse upside down in the middle of the box, the head of the mouse was about 5 cm from the bottom of the hanging tail box. Observe for 6 minutes and record the time of immobility after 4 minutes. The criterion for mobility is that the animal stops struggling, its body hangs vertically and stays still. The experiments were fasted for 12 h before the experiment and performed 30 min after intragastric administration.

1. **Forced swimming test (FST) in mice**

The dose-effect relationship of GW043 in the mouse FST was investigated through a series of two experiments. In the experiment, Each mouse (20-25 g) was placed individually into a transparent glass container filled with 10 cm of water (temperature of 23-25 °C). Total duration of the session was 6 minutes (the first 2 minutes of pre-test followed by a 4-minute test), and the immobility time, regarded as a measure of behavioral despair/depressive-like behavior, was scored during the last 4 minutes. All trials were recorded by a video camera, and posteriorly analyzed by an experimenter blind to the treatment/microbiome status.

1. **Forced swimming test (FST) in rats**

36 naïve SD rats were randomly divided into 4 groups (n = 9 /group): control group, 10 mg/kg fluoxetine, 0.01 mg/kg GW043, 0.02mg/kg GW043. The drug was administered via gavage 60 minutes prior to the initiation of the experiment. The procedure consisted of two phases, the pretest phase and the test phase, with the use of identical cylinders and conditions (20 cm in diameter, 60 cm in height, containing 33 cm of water, temperature maintained at 25 °C). At pretest period, the rats were placed in the cylinder alone for 15 min of swimming; after 24 h, the rats were placed in the same device for 5 min, a period designated as the test period. The activity of the rats during the test was recorded with a video camera and the duration of immobility (defined as above), swimming (active horizontal movement, i.e. around or through the cylinder) or climbing (upward movement with the front paws pointing towards the cylinder wall) was measured over 5 min. The drug was administered orally 1 h before the test.

1. **Results**
2. **The immobility time of TST in mice**

Supplementary figure 1A illustrates that GW043, when administered at doses of 0.01 mg/kg, had no significant effect on the immobility time of TST in mice(*p*＞0.05). The administration of fluoxetine (10 mg/kg) led to a notable reduction in the immobility time of the mice in the TST (F_2, 27_ = 2.472, *p*＜0.05).

1. **The immobility time of FST in mice**

As shown in Supplementary figure 1B, at doses of 0.01mg/kg and 0.02mg/kg, administration of 0.02mg/kg GW043 resulted in a significant reduction in immobility time in FST mice. The administration of fluoxetine (10 mg/kg) led to a notable reduction in the immobility time of the mice in the FST(F_3, 35_ = 5.235, *p*＜0.05).

At doses of 0.1mg/kg-10.0 mg/kg, GW043 resulted in a significant reduction in immobility time in FST mice. The administration of fluoxetine (10 mg/kg) led to a notable reduction in the immobility time of the mice in the FST(F_4, 53_ = 6.372, *p*＜0.001).

1. **The immobility time of FST in rats**

As shown in Supplementary figure 1C, at doses of 0.01mg/kg and 0.02mg/kg, administration of 0.02mg/kg GW043 resulted in a significant reduction in immobility time in FST rats. The administration of fluoxetine (10 mg/kg) led to a notable reduction in the immobility time of the rats in the FST(F_3, 32_ = 3.106, *p*＜0.05).
